# Perioperative Risks and Outcomes in Asian American Patients with Type 2 Diabetes Mellitus and/or Metabolic Syndrome: a Systematic Scoping Review

**DOI:** 10.1007/s40615-025-02344-6

**Published:** 2025-03-05

**Authors:** Catherine P. Marudo, Vikasni Mohan, Sanjukta Dutta, John M. Reynolds, Aisha Khan, Sabrina N. Taldone, Eugene S. Fu

**Affiliations:** 1 University of Miami Miller School of Medicine, Miami, FL USA; 2 Louis Calder Memorial Library, University of Miami Miller School of Medicine, Miami, FL USA; 3 Department of Anesthesiology, University of Miami Miller School of Medicine, 1400 NW 12th Ave Suite 4022, Miami, FL 33136 USA; 4 Department of Internal Medicine, University of Miami Miller School of Medicine, Miami, FL USA

**Keywords:** Type 2 diabetes mellitus, Metabolic syndrome, Asian American

## Abstract

Asian Americans (AA) have an increased risk of developing type 2 diabetes mellitus (T2DM) and metabolic syndrome (MetS) compared to non-Hispanic White Americans, yet over half of AA patients with T2DM are underdiagnosed or untreated. Surgical stress, known to exacerbate hyperglycemia in T2DM, is also associated with increased morbidity and mortality. Thus, AA patients may be at elevated risk of experiencing poor outcomes following surgery. This review aims to summarize the available literature on the perioperative (defined as before, during, and after surgery) risk and outcomes of T2DM in AA surgical patients and identify specific knowledge gaps. A scoping review protocol was developed in accordance with PRISMA guidelines. Medline, Embase, Web of Science, Scopus, and Cochrane CENTRAL were comprehensively searched for publications without language or date limits on perioperative management of undiagnosed and diagnosed T2DM and/or MetS in AA. Inclusion criteria included full-text studies conducted in the United States (U.S.), specified AA with T2DM and/or MetS as a study population, and focused on perioperative considerations or clinical outcomes. Search results yielded 862 articles imported into Covidence for title, abstracts, full-text screening, and data extraction. Fifteen publications were identified for full review: 13 (86.6%) retrospective cohort study articles, 1 (6.6%) review article, and 1 (6.6%) randomized controlled trial. These articles represented 2,494,987 total patients and 38,440 aggregate Asian American patients (1.5%). Notable findings amongst studies included (1) higher T2DM rates among AA compared to other racial/ethnic groups, (2) diagnosis variations among AA ethnic subgroups, (3) and conflicting findings on postoperative complications in AA. This review highlights knowledge gaps in our current understanding of disparities regarding perioperative risks and outcomes of AA surgical patients with T2DM and/or MetS. There is a need for stronger research methodologies to guide evidence-based recommendations regarding the perioperative risks and optimal management of this patient population.

**Supplementary Information:**

The online version contains supplementary material available at 10.1007/s40615-025-02344-6.

## Introduction

According to the United States (U.S.) Census Bureau, the population of Asian Americans (AA) nearly doubled between 2000 and 2019 and is projected to surpass 46 million by 2060, making Asian Americans the largest immigrant group by mid-century and the fastest-growing racial or ethnic group in the USA [1, 2] AA are at higher risk of developing type 2 diabetes mellitus (T2DM) and metabolic syndrome (MetS) compared to non-Hispanic White Americans (NHW) [3, 4]. However, more than half of AA patients with T2DM are underdiagnosed, putting them at increased potential risk of perioperative complications due to stress hyperglycemia during and after surgery [5]. In AA, T2DM and MetS diagnoses are higher compared to other ethnic and racial groups despite the lower mean body mass index (BMI) in the AA population [3, 4]. The prevalence of T2DM is about 21% among AA, more than twice that in NHW patients [6]. The magnitude of MetS—characterized by truncal obesity, insulin resistance, altered lipid levels, and hypertension—has also been unusually high in East and South Asians compared to NHW patients [7].

The increased risk of T2DM and MetS in AA has been attributed to a combination of physiologic and environmental factors [6, 8]. Major surgical stress induces insulin resistance, resulting in stress hyperglycemia [9]. Perioperative hyperglycemia can lead to immune dysfunction, endothelial dysfunction, coagulopathies, and extended hospital stays following surgery [5]. Impaired prolonged fasting glucose levels are also associated with the development of micro- and macro-vascular complications, which can begin to occur before diagnosis of T2DM and/or MetS [10]. These complications include cardiovascular disease, cerebrovascular disease, retinopathy, neuropathy, and nephropathy [10]. Numerous studies have demonstrated a clear association between perioperative hyperglycemia and T2DM with increased morbidity-related outcomes and increased length of hospital stay [5]. Perioperative risk is higher in patients with undiagnosed T2DM, a diagnosis more common in AA patients [4, 5]. Undiagnosed T2DM is associated with an up to three times increased risk of death and can present significant long-term effects or surgical complications [11]. Patients who develop MetS have a five-fold increased risk of developing T2DM [12]. MetS poses specific perioperative challenges due to its broad definition and varied presentation, including obesity, dyslipidemia, hyperglycemia, insulin resistance, and hypertension [4].

Given increased risk and higher rates of undiagnosed T2DM and/or MetS in AA, this systematic scoping review aims to summarize the available literature on perioperative management of T2DM and perioperative clinical outcomes in AA surgical patients and identify specific knowledge gaps. The objective of this review is to understand how the increased risk of T2DM and MetS in AA patients impacts the perioperative outcomes to better understand the unique needs of AA patients with T2DM and MetS during the perioperative period. By assessing risks and outcomes, clinicians can gain improved insight into the perioperative considerations and management of AA patients undergoing surgical procedures.

In this paper, we define perioperative management as the management of AA patients with T2DM and MetS before, during, and after surgery. Perioperative complications include unexpected problems that occur before, during, and after surgery, such as bleeding and wound infections. Perioperative outcomes are defined as the overall result of the surgery, including both expected and unexpected results. These terms were used to understand how assessing risk and outcomes may influence the perioperative considerations and management of diabetes of AA patients with T2DM and MetS.

## Methods

### Methodology and Sources

This scoping review was conducted with guidance from the Joanna Briggs Institute (JBI) Manual for Evidence Synthesis and reported in accordance with the Preferred Reporting Items for Systematic Reviews and Meta-analyses Extension for Scoping Reviews (PRISMA-ScR) [13, 14]. The study protocol was registered at the Open Science Framework [15]. This scoping review was registered through the Open Science Framework (OSF) and can be found using DOI 10.17605/OSF.IO/S4J6M or at https://osf.io/s4j6m/.

### Search Strategy

The search strategy was developed by an academic health science librarian (J.R.) in consultation with the research team leaders (C.M. and E.F.). It was reviewed by another medical librarian using the Peer Review for Electronic Search Strategies (PRESS) tool [16]. The search strategy was written for Ovid Medline and translated using each database’s syntax, controlled vocabulary, and search fields. Search terms pertaining to the aims of the study regarding perioperative outcomes in AA with T2DM and/or metabolic syndrome were used. MeSH terms, EMTREE terms, and text words were used for “Type 2 diabetes mellitus,” an extensive range of postoperative complications, “Asian Americas,” and their synonyms. Examples of search terms used to encompass AA patients included the following: Chinese, Indian, Japanese, Filipino, Korean, Malaysian, Bangladeshi, Thai, Vietnamese, Laotian, and their qualifiers. We searched Medline (Ovid, MEDALL), Embase (Elsevier, Embase.com), Cochrane CENTRAL (Cochrane Library, Wiley), Scopus (Elsevier), and the Web of Science platform (Clarivate: Science Citation Index Expanded, Social Sciences Citation Index, Arts & Humanities Citation Index, Conference Proceedings Citation Index-Science, Conference Proceedings Citation Index-Social Science & Humanities, Emerging Sources Citation Index, Preprint Citation Index, ProQuest Dissertations & Theses Citation Index, SciELO Citation Index). A comprehensive but unvalidated filter was used to limit the results to studies on AA. The Medline search strategy was adapted for other databases in part with the use of the Institute for Evidence Based Healthcare’s Polyglot Search translator [17]. No language, date, or other limits were applied. All databases were searched on February 9, 2024. For full search strategies, see Supplementary Materials 1. All database records were downloaded to EndNote 20 and uploaded to Covidence web-based software for deduplication, screening, and full-text evaluation [18, 19]. Covidence is a web-based collaboration software platform that streamlines the production of systematic and other literature reviews. No citation chaining was performed [19]. The studies included in systematic reviews on related topics were also screened. The Retraction Watch database and journal websites were checked for retractions of included studies. All included studies can be found in Table 1. Full citations excluded studies with reason for exclusion can be found in Appendix 1. The included studies and their outcomes are summarized in Tables 1 and 2, respectively.

**Table 1:** Summary of all papers included in the review

Study ID	Aim of study	Type of surgery	Primary outcome(s)	Study design	Key findings	# AA participants	# non-AA participants	# total participants
Wong et al. (2015)	Delineate the impact of obesity using population-based registry data	Liver transplant	Obesity rates, post-transplant survival	Retrospective cohort	There was a greater proportion of NHW patients in the BMI ≥ 30 kg/m2 group and a greater proportion of AA patients in the BMI < 30 kg/m2 group. Although there was a significantly greater prevalence of DM in the ≥ 30 kg/m2 group compared with the BMI < 30 kg/m2 group, a higher proportion of Hispanics and AA with DM compared to NHW. When evaluating differences in post-transplant survival by race/ethnicity, Blacks had significantly worse survival, whereas Hispanics and AA had significantly better survival when compared to NHW	2609	54,646	57,255
Pennington et al. (2020)	Determine whether strict perioperative glycemic control independently influences SSI risk outside of well-known factors such as diabetes and chronic steroid use	Spine	SSI	Retrospective review	Postoperative hyperglycemia and poor postoperative glucose control are independent risk factors for SSI following surgery for degenerative spine disease. Among high-risk DM patients, strict perioperative glucose control may decrease the risk of SSI. However, the study did not stratify outcomes by race, specifically AA vs. non-AA	3	114	117
Mamidi et al. (2021)	Determine the effect of DM (IDDM and NIDDM) on surgical outcomes in patients following tonsillectomy	Tonsillectomy	Postoperative complications, prolonged hospitalization, unplanned readmissions	Retrospective cohort	DM patients are at a heightened risk for complications following tonsillectomy. However, the study did not stratify outcomes by race/ethnicity to discuss differences in AA vs. non-AA	707	27,053	27,760
Johnstone et al. (2024)	Elucidate disparities among patient demographics by identifying predictors of complications and postoperative outcomes among different racial/ethnic patients undergoing breast reconstruction	Breast reconstruction	Postoperative complications. Seroma, deformity or dehiscence, fat and tissue necrosis	Retrospective analysis	Black ethnicity (relative to NHW), age, autologous reconstruction, hypertension, type II diabetes mellitus, and tobacco use were independent predictors of increased complication likelihood. In contrast, Asian ethnicity was associated with a reduction in the likelihood of a postoperative complication. AA patient records also had a reduced likelihood of postoperative seroma, breast reconstruction deformity and dehiscence, and the likelihood of fat and tissue necrosis	2744	101,970	104,714
Williams et al. (2010)	Determine the frequency of aortic dissection complications in the Society of Thoracic Surgeons database and the outcomes of patients who suffer intraoperative aortic dissection	Cardiac	Aortic dissection	Retrospective cohort	Preoperative characteristics such as Asian race (independent of body surface area), treatment with steroids, peripheral vascular disease, and age greater than 60 were associated with an increased risk of aortic dissection. Patients with DM were less likely to suffer an acute dissection as a complication	29,032	2,190,959	2,219,991
Unalp-Arid et al. (2023)	Examine gallbladder and biliary tract mortality predictors with linked mortality data and gallstone disease prevalence trends and associations	Gallbladder	Gallbladder and biliary tract mortality	Retrospective cohort	Older age and female sex were associated with higher odds of diagnosed gallstone disease or gallbladder surgery, and NHB and AA had lower odds	1120	8112	9232
Lockridge et al. (2021)	Assess rates of new-onset diabetes after transplant according to age, ethnicity, body weight, BMI, rejection, and prednisone dosing among kidney transplant recipients	Renal transplant	NODAT	Retrospective cohort	After adjusting for common NODAT risk factors, the analysis indicated that age and corticosteroid dose in the AA population [adjusted for lower body weight, BMI] significantly increased the risk of NODAT. In the present study, 34% of AA renal transplant recipients developed NODAT compared to 16% incidence among non-AA. In multi-variance analysis, despite receiving lower standard doses of protocol corticosteroid daily, AA recipients had a high incidence of NODAT compared to other ethnicities when adjusted for actual body weight. AA received higher doses of corticosteroids (prednisone) than non-AA. Despite the increased rates of NODAT in AA, there was no increased rejection to suggest that a higher dose of immunosuppression contributed to the overall incidence of NODAT	28	235	263
Vanderhoek et al. (2023)	Determine the association between perioperative dysglycemia and 30-day adverse surgical events in pediatric patients undergoing non-cardiac surgery	Pediatric	Adverse surgical events	Retrospective cohort	AA participants were included in the study. However, no specific findings related to AA were reported	220	5190	5410
Margulies et al. (2024)	Assess for racial/ethnic disparities in SUI surgeries, surgical complication differences, and trends over time	Bladder	Postoperative complications, cardiac arrest, hospital admission, UTI, SSI, DVT, sepsis, readmission, wound disruption, blood transfusion	Retrospective cohort	NHB and AA patients had a higher frequency of DM. AA patients were less likely to undergo surgery with general anesthesia than other race/ethnicity groups. AIAN patients had the highest rate of inpatient status (45%), with NHB and AA patients having a higher rate of inpatient status (27% and 26%) than White patients. There were no differences between AA and NHW patients in the rate of sling versus other stress urinary incontinence surgeries	1693	51,640	53,333
Golden et al. (2012)	Provide a scholarly review of the published literature on biological, clinical, and nonclinical contributors to race/ethnic and sex disparities in endocrine disorders and identify current gaps in knowledge as a focus for future research needs	Endocrine, Thyroid, Cesarean section	Prevalence and determinants of disparities in endocrine disorders	Scholarly review	Compared with NHWs, the risk of incident DM was 18% higher in AA, with a significant difference between subgroups. Studies of blood pressure control and hypertension prevalence comparing NHWs to Native Americans and AAs with DM are lacking. Studies indicate lower self-monitoring blood glucose rates among NHBs, Hispanic Americans, and AAs than NHWs. Population-based studies have consistently demonstrated that GDM prevalence is higher in Hispanics, AA, and Native Americans compared with non-NHWs and non-NHBs, with AA experiencing the smallest relative effects of GDM upon macrosomia and cesarean deliveries. Studies suggest that AA has reduced beta-cell function; this represents an additional prevention and treatment target for this population	N/A	N/A	N/A
Dougherty et al. (2021)	Assess for a relationship between immediate preoperative glucose concentrations and postoperative complications	All non-cardiac surgical procedures	Postoperative complications	Retrospective cohort	Preoperative hyperglycemia within 6 h of surgery is a marker of adverse postoperative outcomes. AAs with DM were included as a population in this study, but no specific analyses stratified outcomes by race	15	1759	1774
Valencia et al. (2019)	Assess differences in diabetic outcomes among other ethnic groups, including Hispanic and Asian patient populations	RYGB and LSG	Diabetes remission	Retrospective cohort	Of the 687 DM patients who underwent RYGB or LSG that were analyzed, the majority were NHW (52%), followed by Hispanic (34%), NHB (9%), and AA (5%). Asian race was more positively associated with private insurance (P ¼ 0.0001). Within-group comparisons in all ethnic groups showed significant reductions in body mass index, body weight, fasting insulin, fasting glucose, and HbA1c by six months, but AA patients did not experience further improvement in body mass index or diabetic outcomes at the 12-month visit. Nevertheless, the majority of patients had DM remission by the 12-month postoperative visit (98%, 97%, 98%, and 92% in NHW, Hispanic, NHB, and AA, respectively). This study suggests that additional interventions that support NHB and AA patients with achieving similar metabolic outcomes as NHW and Hispanic patients warrant further consideration	36	651	687
Thawanyarat et al. (2023)	Characterize the effect of time between NAC and mastectomy with immediate reconstruction on postoperative complications	Breast	NAC and mastectomy with immediate reconstruction postoperative outcomes	Retrospective review	Autologous reconstruction, hypertension, type 2 DM, and African American, White, and Hispanic race (relative to Asian) had statistically significant associations with increased complication likelihood	Not reported	Not reported	13,399
Osman et al. (2021)	Assess whether preoperative HbA1c and preoperative blood glucose levels are associated with an increased risk for postoperative infection in diabetic men	Penile	Postoperative infection	Retrospective review	Preoperative blood glucose levels and HbA1c levels are not associated with an increased risk for postoperative infection, revision, or explantation in diabetic men undergoing penile prosthesis implantation. However, the study did not stratify outcomes based on race or ethnicity	200	732	932
Ikramuddin et al. (2015)	Assess outcomes of diabetes control and treatment risks two years after adding RYGB to intensive lifestyle and medical management	Gastric bypass	Adverse events, nutritional deficiencies	Randomized controlled trial	The addition of RYGB to lifestyle and medical management in patients with type 2 DM improved diabetes control, but adverse events and nutritional deficiencies were more frequent. The study included East Asians but did not stratify outcomes by racial/ethnic groups	33	87	120

*NHW Non-Hispanic White; *AA* Asian American; *NHB* Non-Hispanic Black; *BMI* Body mass index; *NODAT* New onset diabetes after transplant; *SSI* Surgical site infection; *DM* Diabetes mellitus; *GDM* Gestational diabetes mellitus; *NAC* Neoadjuvant chemotherapy; *RYGB* Roux-en-Y gastric byoass; *HbA1c* Hemoglobin A1C; *LSG* Laparoscopic sleeve gastrectomy; *UTI* Urinary tract infection; *DVT* Deep venous thrombosis; *AIAN* American Indians and Alaska Natives

**Table 2:** Summary statistics and effect measures of included studies pertaining to Asian participant outcomes

Study ID	Type of surgery	Primary outcome(s)	Study design	Type of Asian	Comparison groups	Summary statistic	Effect measure
Wong et al. (2015)	Liver transplant	Post-transplant survival	Retrospective cohort	Not specified	White, non-Hispanic, Hispanic, Black/African American	Asians = 0.045 of study population	Asians (HR 0.86, 95% CI 0.75–0.97, *p*-value < 0.001)
Pennington et al. (2020)	Spine	SSI	Retrospective review	Not specified	Caucasian, Black/African American, Other: not specified	No specific analysis was done stratifying postoperative outcomes related to T2DM by race	
Mamidi et al. (2021)	Tonsillectomy	Postoperative complications, prolonged hospitalization, unplanned readmissions	Cohort study	Not specified	Caucasian, Black/African American, Native American	No specific analysis was done stratifying postoperative outcomes related to T2DM by race	
Johnstone et al. (2024)	Breast reconstruction	All cause complications outcomes	Retrospective analysis	Not specified	White, non-Hispanic, Hispanic, Black/African American	Asians = 0.026 of study population	OR = 0.77 ([95% C: 0.69, 0.86) *P*-value < 0.001
Williams et al. (2010)	Cardiac	Aortic dissection	Retrospective cohort	Not specified	White, non-Hispanic, Hispanic, Black/African American	Asians = 0.013 of study population	Variable: Asian (%) no aortic dissection (1%) vs. aortic dissection (3%) *P*-value = < 0.001
Unalp-Arid et al. (2023)	Gallbladder	Gallbladder and biliary tract mortality	Retrospective cohort	Not specified	Black/African American, non-Hispanic, White, non-Hispanic, Hispanic, Other: Mexican American	No specific analysis stratifying postoperative outcomes related to T2DM by race	
Lockridge et al. (2021)	Renal transplant	New onset diabetes after transplant	Retrospective cohort	Not specified	Caucasian, Hispanic, Black/African American, Other: other/unknown	Asians = 0.106 of study population	HR = 4.66 [95% CI 1.13–16.23, *p* = 0.02]
Vanderhoek et al. (2023)	Pediatric	Adverse surgical events	Retrospective cohort	Not specified	Black/African American, non-Hispanic, White, non-Hispanic, Other: other/unknown	No specific analysis was done stratifying postoperative outcomes related to T2DM by race	
Margulies et al. (2024)	Bladder	Postoperative complications, cardiac arrest, hospital admission, UTI, SSI, DVT, sepsis, readmission, wound disruption, blood transfusion	Retrospective cohort	Not specified	Black/African American, non-Hispanic, White, non-Hispanic, Hispanic, Native American, Other	Asians = 0.031 of study population	Asian patients had a higher rate of inpatient status (26.28%) than White patients (21.46%)
Golden et al. (2012)	Endocrine, thyroid, cesarean section	Prevalence and determinants of disparities in endocrine disorders	Scholarly review	East, south, southeast, pacific islander	Black/African American, non-Hispanic, White, non-Hispanic, Caucasian, Hispanic, Black/African American, Native American	Not applicable to this study design	
Dougherty et al. (2021)	All non-cardiac surgical procedures	Postoperative complications	Retrospective cohort	Not specified	Caucasian, Hispanic, Black/African American, Other: not specified	No specific analysis was done stratifying postoperative outcomes related to T2DM by race	
Valencia et al. (2019)	RYGB and LSG	Diabetes remission	Retrospective cohort	Not specified	White, non-Hispanic, Hispanic, Black/African American	Asians = 0.052 of study population	At the 12-month visit, 48% of Asian patients had prediabetic fasting glucose or hemoglobin A1c levels, compared to 20% of non-Hispanic white, 19% of Hispanic, and 20% of black patients
Thawanyarat et al. (2023)	Breast	Neoadjuvant chemotherapy and mastectomy with immediate reconstruction postoperative outcomes	Retrospective review	Not specified	White, non-Hispanic, Hispanic, Black/African American	The proportion of Asians making up the study population was not reported	Autologous reconstruction, hypertension, type II diabetes, and African American, White, and Hispanic race (compared to Asian) were significantly associated with higher complication risk
Osman et al. (2021)	Penile	Postoperative infection	Retrospective review	Not specified	Caucasian, Hispanic, Black/African American, Other: not specified	No specific analysis was done stratifying postoperative outcomes related to T2DM by race	
Ikramuddin et al. (2015)	Gastric bypass	Adverse events, nutritional deficiencies	Randomized controlled trial	East	White, non-Hispanic, Hispanic, Black/African American, Native American	No specific analysis was done stratifying postoperative outcomes related to T2DM by race	

*NHW Non-Hispanic White; *NHB* Non-Hispanic Black; *NODAT* New onset diabetes after transplant; *SSI* Surgical site infection; *DM* Diabetes mellitus; *GDM* Gestational diabetes mellitus; *RYGB* Roux-en-Y gastric bypass; *LSG* Laparoscopic sleeve gastrectomy; *UTI* Urinary tract infection; *DVT* Deep venous thrombosis

### Exclusion Criteria

Articles not conducted in the U.S. or did not address the surgical outcomes or pre-, intra-, and/or postoperative considerations of T2DM and/or MetS in AA patients were excluded. To keep our search broad, we considered all article types in our search. Given our research team’s language and resource limitations, articles not published in English for which we could not find a qualified reader and articles without full text, such as conference abstracts with insufficient data, were also excluded.

### Study Selection

The titles and abstracts of articles were screened and included if they contained the terms type 2 diabetes mellitus, metabolic syndrome, Asian American, Asian ethnic subgroups, perioperative (including pre-, intra-, and postoperative) complications, and synonyms. Two reviewers independently screened abstracts using the inclusion and exclusion criteria, and a third reviewer resolved discrepancies between reviewers. A similar process was conducted for the full-text screening phase.

### Data Extraction

Covidence was used to extract data from full-text articles that met inclusion criteria. Two reviewers independently extracted data from the papers, with a third reviewer resolving conflicts. Data extracted for each article included the title, year of publication, author, corresponding author’s email, study design, perioperative conditions reported, subgroup of AA identified, and key findings. In U.S. studies, patient populations described as “Asian” were assumed to be “Asian Americans.” However, limited studies clearly defined their “Asian” patient population as Asian Americans, Asian immigrants, or a specific Asian ethnic subgroup. Missing information was noted as “not applicable.” A quality assessment form based on the JBI Critical Appraisal Tool for systematic reviews was completed for each paper (Table 3).

**Table 3:**
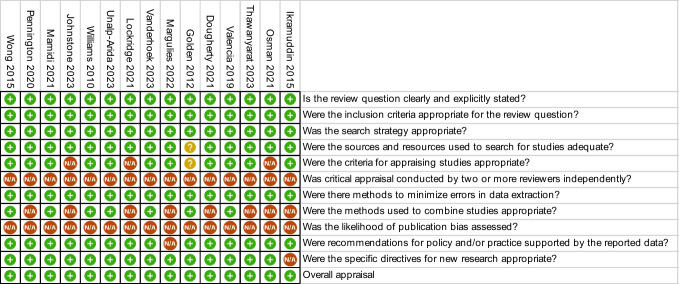
Quality assessment summary

Criteria based on Joanna Briggs Inst itute (JBI) crit ical appraisal checklist

Yes, or include

Unclear

Not applicable

## Results

### Descriptive Data

**Fig. 1:**
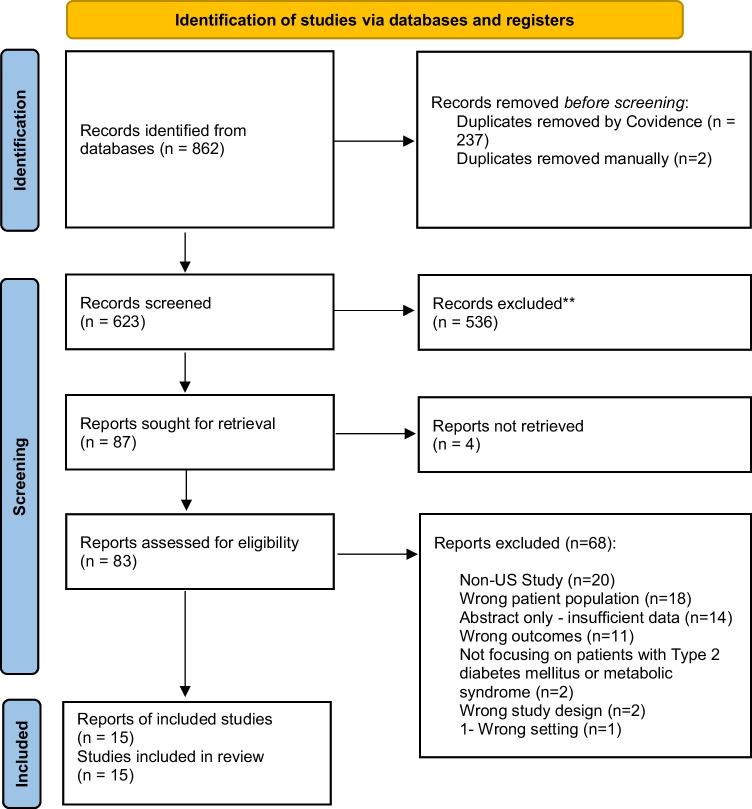
The PRISMA (Preferred Reporting Items for Systematic Reviews and Meta-Analyses) flowchart demonstrates the full scoping review process, from initial search to abstract screening and full-text assessment

The systematic search produced 862 records. After 237 duplicates were removed by Covidence software and two records were removed manually, 623 titles and abstracts were screened, and 536 were excluded. The full text of 87 articles were assessed for eligibility. Full texts of 4 studies could not be found, and those records were thus excluded. Additionally, Molnar et al. (2011) stated the use of case mixed-adjusted models that included recipient race/ethnicity (including “Asians”) in statistical analysis, but no specific data regarding Asian participants included in the study was published [20]. The study team reached out to the corresponding author of Molnar et al. (2011) on 3/31/2024 to request the missing data but has not received a response. Therefore, this study was subsequently excluded because of insufficient data. Overall, 15 papers were included based on the predetermined criteria (described above), with studies focusing on AA in the perioperative period [21–35]. The remaining 68 articles were excluded; the reasons are summarized in Fig. 1. Table 1 summarizes papers included in the final data extraction. Study designs included 13 (86.6%) retrospective cohort study articles, 1 (6.6%) review article, and 1 (6.6%) randomized controlled trial for full review. These articles represented 2,494,987 total patients and 38,440 aggregate Asian American (AA) patients (1.5%). The range of participants with T2DM and/or MetS across included studies that reported a participant count between 3 and 29,032. The surgical specialties represented included gastric, hepatobiliary, otolaryngology, spine, breast, cardiac, renal, urology, endocrine, pediatric, and obstetric.

### Data Synthesis

The results of our data extraction highlight three common themes among the included studies: higher T2DM rates among AA surgical patients compared to other racial/ethnic groups, variations among Asian American ethnic subgroups, and perioperative risks and complications in AA related to T2DM,

#### Theme 1: Higher T2DM Rates Among AA Surgical Patients Compared to Other Racial/Ethnic Groups

Asian Americans not only have higher rates of T2DM when compared to other racial and ethnic groups but the increased prevalence of T2DM is found at lower BMIs in AA when compared to other groups [3, 36]. AA surgical patients are also more likely to have a lower BMI (BMI < 30 kg/m2) than NHW participants (BMI > 30 kg/m^2^) despite higher rates of T2DM [21]. Additionally, Golden et al. (2012) found that compared with NHW participants, the risk of incident T2DM was 18% higher in AA surgical patients, greatly impacting overall health and wellbeing [35].

#### Theme 2: Variations Among Asian American Ethnic Subgroups

Variations in T2DM prevalence and metabolic risk factors between ethnic subgroups were common among papers included in this scoping review and must be considered when discussing perioperative risks. In the study by Golden et al. (2012), data from the National Health Interview Survey (NHIS) was used to highlight significant ethnic variations in the prevalence of T2DM within AA surgical patients, with the highest prevalence among Asian Indians and Filipinos (10–15%) and the lowest prevalence among Koreans, Vietnamese, and Chinese (3–7%) in the U.S [35]. Of all the extracted studies, only Golden et al. and Ikramuddin et al. stratified AA by sub-ethnic subgroups and looked at the sub-ethnic group of East Asians, respectively [27, 35]. Gordon et al. (2019) analyzed 1.4 million adult electronic health records in Northern California [37]. They found significant differences in health burden between AA ethnic subgroups, confirming that reporting statistics for an aggregated AA racial group masks meaningful differences in AA ethnic subgroup health [37]. These differences may contribute to conflicting results of studies seeking to understand AA risk for perioperative T2DM complications.

#### ThemeAQ 3: Perioperative Risks and Complications in AA Related to T2DM

Perioperative complications were the focus of this paper. However the studies found focused on postoperative complications, and there was a lack of papers addressing complications during the preoperative and interoperative periods. Five studies from this scoping review (Table 1) confirmed the increased incidence of T2DM in AA compared to NHW [21, 25, 29, 31]. However, they found paradoxically lower rates of perioperative complications in AA despite T2DM being associated with increased perioperative complications [21, 25, 29, 31]. It is important to note that perioperative complications can encompass many different conditions and can be influenced by multiple confounding factors.

Williams et al. (2010) found that Asian race, independent of body surface area or BMI, was a predisposing risk factor for aortic dissection as a complication of cardiac surgery [30]. In a study by Margulies et al. (2024), AA had the highest rates of inpatient status after urinary incontinence surgery compared to any other racial or ethnic group [34]. However, the reasons behind differences in inpatient status were not discussed. In a study by Lockridge et al. (2021), nondiabetic AA had higher rates of new-onset diabetes after kidney transplant compared to Caucasians [32]. This study found that even after accounting for lower average body weights and BMIs in the AA cohort, Asian ethnicity predisposed patients to developing T2DM after kidney transplant [32].

## Discussion

The findings of our literature review show a higher incidence of T2DM and lower BMI in AA in the U.S. than in NHW, which is consistent with the existing literature. Similar statistics have been reported by the U.S. Department of Health and Human Services, which reports that AAs are 40% more likely to be diagnosed with T2DM than NHWs [38]. These disparities are compounded, as T2DM can lead to other health conditions, including heart disease, neuropathy, nephropathy, and retinopathy, and ultimately increase morbidity and mortality. While many factors can increase the risk of poor surgical outcomes and surgical site infections, it is important to consider AAs as having an increased propensity for developing T2DM. Thus, it is important to consider screening AA surgical patients for T2DM due to their higher rates of T2DM and potentially increased risk of surgical complications [39]. However, more research is needed to determine when to screen AA for T2DM and/or MetS in the perioperative period (possible criteria include elevated BMI, increased waist circumference ratio, elevated BP, dyslipidemia, etc.).

Asian Americans as a demographic encompass a vast and diverse group of individuals. These include individuals of South Asian (e.g., Indian, Pakistani), East Asian (e.g., Chinese, Japanese, Korean), and Southeast Asian (e.g., Filipino, Thai, Vietnamese) descent who are born in and/or residing within the U.S [35]. According to the Pew Research Center, Chinese Americans make up the largest of these ethnic subgroups, at 24% of all AA [40]. They are followed closely by Indian Americans and Filipino Americans, who comprise 21% and 19% of all AA, respectively [40]. A 2022 study in California found that within AA ethnic groups, Filipino and South Asian Americans had considerably higher diabetes prevalence than Chinese Americans [41].

Although the literature review results were consistent with existing literature showing Filipino, Japanese, and South Asian groups having the highest prevalence of T2DM across all AA ethnic subgroups [41–43], there was still a significant knowledge gap in the literature regarding the impact of T2DM and/or MetS on perioperative risks and outcomes among the different AA ethnic subgroups. A study of the 2009 California Health Information Survey (CHIS) data showed that Native Americans, non-Hispanic Blacks, and Hispanic Americans had a higher overall age-adjusted prevalence of T2DM compared to AA overall [43]. However, when separated by ethnic subgroups, Filipino (15.8%) and Japanese American (11.8%) men were found to have a higher age-adjusted prevalence of T2DM than both non-Hispanic Black and Hispanic American men [43]. Thus, studies involving AA can calculate different T2DM prevalence rates depending on the composition of specific AA subgroups within the broader category of AA. As reported in a previous study of AA subgroups, aggregating AA into a single category can potentially minimize the disease prevalence among high-risk subgroups while overestimating the risk among low-risk subgroups within the same demographic [37].

Unlike studies that cited T2DM as a risk factor for complications [30], T2DM was found to be a protective factor against aortic dissection [44, 45]. The causal associations based on race found in studies like Williams et al. and Margulies et al. may also be limited because they make assumptions about race without understanding what race represents in a specific context (i.e., the impact of social determinants of health on the outcome measures) [44, 45]. Social determinants of health have often been “confounders” in the cause-effect hypothesis of race and increased disease prevalence. The Lockridge study also poses further research questions regarding why race contributes to increased T2DM development after a successful renal transplant and what this means for clinical practice and risk–benefit patient counseling.

Conflicting findings in the literature indicate a gap in the current understanding of T2DM complications in AA surgical patients, and further research should focus on the causes of disparities in AA with comorbid conditions that may predispose to perioperative complications. One possible explanation of these conflicting findings is the variability in T2DM prevalence and metabolic profiles between AA ethnic subgroups, as discussed previously. With varying ethnic subgroup aggregations across the U.S., AA in California may exhibit a significantly different metabolic profile than AA in other parts of the U.S., leading to conflicting, even paradoxical, findings between the two groups.

## Strengths

To our knowledge, this scoping review is the first to summarize the existing literature on surgical outcomes and the pre-, intra-, and postoperative considerations of T2DM and MetS in Asian American patients in the U.S. The scoping nature of this review article provides a comprehensive overview of the available literature, identifies gaps in the existing body of research, and explores an emerging topic in the perioperative space to explore racial and ethnic disparities, why they may exist, and provide evidence-based practice guidelines to reduce disparities. Furthermore, articles were double-independently screened and appraised for overall quality to minimize bias. Understanding disparities in perioperative outcomes in this population is essential to guiding future research, optimizing clinical practice, and targeting interventions that will benefit this patient population. Research in this area becomes increasingly important as AAs are the fastest-growing racial-ethnic group in the U.S. and as the burden of T2DM diagnosis disproportionately increases among these patients compared to NHW Americans.

## Limitations

Despite the thoroughness of this scoping review, including a high-quality search of multiple databases, double-independent screening of the literature search results, and a quality assessment of included studies, the likelihood of publication bias was not assessed. This paper also explicitly focused on studies conducted in the U.S. and did not include non-English papers. Many of the studies excluded from the final data extraction were Asians studied in different parts of the world, leading to relatively few studies focusing solely on AA in the U.S. The exclusion of international articles may introduce bias and limit the generalizability of our review. Furthermore, while this review focused on AA with T2DM and/or MetS in the U.S., studies often did not specify whether the population studied was AA or Asian immigrants in the U.S. Additionally, there were also relatively few studies that focused on all elements of the perioperative period, suggesting that further research is needed particularly the preoperative and intraoperative considerations for this population. As previously mentioned, among the extracted studies, only Golden et al. and Ikramuddin et al. stratified AA by sub-ethnic subgroups and looked at the sub-ethnic group of East Asians, respectively [27, 35]. The lack of stratification by ethnic subgroup among AA limits the current understanding of how T2DM and/or MetS impacts the perioperative risks and outcomes of AA.

There was also significant heterogeneity in the data presented by the studies due to their different study types and different outcomes of focus, making it difficult to draw meaningful associations or conclusions. The absence of prospective cohort studies and only one randomized controlled trial in the current literature further diminishes the ability to provide evidence-based recommendations on how providers should consider the perioperative management and optimization of surgical outcomes of AA with T2DM or MetS. Thus, more high-quality research is needed to better understand the risk of perioperative complications in AA surgical patients with T2DM and/or MetS and to guide evidence-based practice recommendations in the perioperative setting.

## Conclusions

In conclusion, this scoping review found that the underdiagnosis of T2DM among Asian American patients is under-recognized in health disparities research in general and specifically in the surgical literature. There are limited studies assessing the perioperative risks and management of AA surgical patients with T2DM and/or MetS, highlighting significant gaps in the current understanding of perioperative risks and outcomes in this population. Although we found consistencies amongst studies regarding increased T2DM rates among AA compared to other racial/ethnic groups, there were variations in T2DM rates among AA ethnic subgroups and conflicting findings comparing postoperative complications of T2DM and/or MetS between AA and other racial groups. Lastly, U.S.-specific studies primarily investigating differences in perioperative complications of T2DM and/or MetS between AA and other racial groups were limited. This review highlights gaps and inconsistencies in the current understanding of perioperative risks and outcomes in AA patients with T2DM and/or MetS, emphasizing the need for further epidemiological and observational studies to improve screening guideline recommendations and health data accuracy for this patient population. Additional research would benefit from stratifying AA into ethnic subgroups to investigate disparities in postoperative outcomes between subgroups. Ultimately, further research on the impact of diagnosed and undiagnosed T2DM in AA will allow anesthesiologists and perioperative physicians to better understand and manage perioperative risk in this patient population.

## Supplementary Information

Below is the link to the electronic supplementary material.

Supplementary file1 (DOCX 57 kb)

Supplementary file2 (DOCX 25 kb)

## Data Availability

The authors confirm that the data supporting the findings discussed in the article are available as part of the article and its Supplementary materials. No additional source data are required.
