# Supplementary material for: Perioperative Risks and Outcomes in Asian American Patients with Type 2 Diabetes Mellitus and/or Metabolic Syndrome: a Systematic Scoping Review
Source: J Racial Ethn Health Disparities. 2025 Mar 5;13(2):1417–33. doi: 10.1007/s40615-025-02344-6 (PMC12966264; doi:10.1007/s40615-025-02344-6)
Supplement: Supplementary file 1 — Supplementary file1 (DOCX 57 kb) [file 40615_2025_2344_MOESM1_ESM.docx]

**Supplementary Materials 1: Database Search Strategies**

**Perioperative considerations and outcomes in Asian American patients with Type 2 diabetes mellitus and/or metabolic syndrome: A scoping review**

**Ovid MEDLINE(R) ALL <1946 to February 08, 2024>
Searched February 9, 2024.
71 records retrieved.**

| 1 | exp Diabetes Mellitus, Type 2/ | 177159 |
| --- | --- | --- |
| 2 | (Diabet* adj2 (adult-onset or insulin-independent* or ketosis-resistant or latent* or matur*-onset or non-insulin-dependent or slow-onset or stable or type-2 or type-II)).ti,ab,kf. | 210168 |
| 3 | (DM-2* or DM2* or MODY* or NIDDM* or T2D* or T2DM*).ti,ab,kf. | 66449 |
| 4 | Metabolic Syndrome/ | 39150 |
| 5 | ((metabolic* or cardiometabolic or cardio-metabolic* or dysmetabolic* or dys-metabolic* or insulin-resistan* or reaven*) adj3 syndrom*).ti,ab,kf. | 71725 |
| 6 | (a1c or hb-a1c or haemoglobin-a1c or haemoglobin-a-1c or haemoglobin-aic or hba-1c or hba1c or hemoglobin-a1c or hemoglobin-a-1c or hemoglobin-aic).ti,ab,kf. | 65661 |
| 7 | (glucosaemi* or glucosemi* or glycaemi* or glycemi* or hyperglycaemi* or hyperglycemia* or hyper-glycemia* or hyperglycaemi* or hyper-glycaemi* or hyperglucemi*).ti,ab,kf. | 134685 |
| 8 | or/1-7 | 421093 |
| 9 | (Intraop* or Intra-op* or intrasurg* or intra-surg*).ti,ab,kf. | 195705 |
| 10 | ((oper* or surg*) adj2 (durat* or length* or time*)).ti,ab,kf. | 144862 |
| 11 | Postoperative Period/ or Postoperative Care/ | 116409 |
| 12 | (postop* or post-op* or postsurg* or post-surg* or post-proced*).ti,ab,kf. | 811215 |
| 13 | ((operat* or surg* or proced*) adj7 sequelae*).ti,ab,kf. | 3962 |
| 14 | exp Perioperative Care/ or exp Perioperative Period/ or Perioperative Medicine/ | 255985 |
| 15 | (periop* or peri-op* or perisurg* or peri-surg*).ti,ab,kf. | 140607 |
| 16 | exp Perioperative Nursing/ | 13982 |
| 17 | Anesthesia Recovery Period/ | 5565 |
| 18 | exp Anesthesia/ or exp Conscious Sedation/ or exp Deep Sedation/ or exp Neuromuscular Blockade/ or exp Preanesthetic Medication/ | 220403 |
| 19 | (anesthe* or anaesthe* or cryoanaesthe* or cryo-anaesthe* or cryoanesthe* or cryo-anesthe* or electro-anaesthe* or electroanasthaesia or electroanesthe* or electro-anesthe* or neuro-anaesthe* or neuroanasthaesia or neuroanesthe* or neuro-anesthe* or neuroleptanaesthe* or neuro-leptanaesthe* or neuroleptanesthe* or neuro-leptanesthe*).ti,ab,kf. | 438881 |
| 20 | (block* adj3 (autonomic or cauda or caudal or cervical or conduction or dural or epidural or extradural or ganglion or intercostal or lumbar or nerve or neurogenic or neuromuscular or paracervical or peridural or 'QL ' or quadratus lumborum or retrobulbar or retroocular or spinal or stellate-ganglion or subarachnoid or TAP or "transverse abdominal plane")).ti,ab,kf. | 49677 |
| 21 | (postanesthes* or post-anesthes* or postanaesthes* or post-anesthaes* or (recover* adj (room* or ward* or period*)) or PACU).ti,ab,kf. | 24589 |
| 22 | Intraoperative Complications/ or Blood Loss, Surgical/ or Intraoperative Awareness/ or Malignant Hyperthermia/ | 57298 |
| 23 | ((surg* or operat*) adj2 (advers* or injur* or complicat* or blood-loss or hemorrhag* or haemorrhag*)).ti,ab,kf. | 99438 |
| 24 | ((anesthesi* or anesthaesi* or malignan*) adj4 (hypertherm* or hyperpyrex*)).ti,ab,kf. | 4676 |
| 25 | exp Postoperative Complications/ | 620337 |
| 26 | exp Surgical Procedures, Operative/ae, co [Adverse Effects, Complications] | 539670 |
| 27 | ((afferent* or efferent*) adj (loop* or limb*) adj3 (syndrome* or disease* or obstruct*)).ti,ab,kf. | 515 |
| 28 | ((anastomosis* or anastomotic*) adj3 (dehiscen* or leak* or rupture*)).ti,ab,kf. | 14530 |
| 29 | (((breast* adj2 cancer) or mastectom*) adj3 (lymphedem* or lymphoedem*)).ti,ab,kf. | 1759 |
| 30 | (cornea* adj endothel* adj2 (loss* or damag* or injur*)).ti,ab,kf. | 854 |
| 31 | coronary-subclavian steal.ti,ab,kf. | 229 |
| 32 | ((delay* adj2 (awake* or conscious* or (emergenc* adj2 anesth?es*))) or (residual adj2 (block* or curarizat*))).ti,ab,kf. | 1713 |
| 33 | ((emerg* or post-an?esth* or postan?esth*) adj (deliri* or agitat* or excit*)).ti,ab,kf. | 1146 |
| 34 | (failed back surg* or ((failed-back or post-laminectom* or postlaminectom*) adj2 syndrome*) or FBSS).ti,ab,kf. | 1513 |
| 35 | Graft Rejection/ | 64816 |
| 36 | ((graft* or allograft* or transplant* or flap*) adj2 (occlusion* or occlude* or restenosi* or disease* or disorder* or dysfunct* or fail* or infect* or necros* or reject* or vasculopath*)).ti,ab,kf. | 86205 |
| 37 | ((cicatr* or incision* or scar*) adj3 hernia*).ti,ab,kf. | 6102 |
| 38 | (pseudomeli* or pseudo-meli* or (phantom adj3 (limb* or pain* or sensation*))).ti,ab,kf. | 2779 |
| 39 | (cholecystectom* or post-cholecystectom* or postcholecystectom* or choledochoduodenostom* or post-choledochoduodenostom* or postcholedochoduodenostom* or sump).ti,ab,kf. | 36271 |
| 40 | (postgastrectom* or post-gastrectom* or stomach-resect* or dumping*).ti,ab,kf. | 6928 |
| 41 | (endoleak* or endo-leak* or perigraft-leak*).ti,ab,kf. | 6560 |
| 42 | ponv.ti,ab,kf. | 2976 |
| 43 | (postcommissurotom* or postpericardiotom* or post-commissurotom* or post-pericardiotom*).ti,ab,kf. | 464 |
| 44 | ((prosthesis* or prosthetic*) adj3 (fail* or complicat* or durab* or loos* or migrat* or infect* or surviv*)).ti,ab,kf. | 14444 |
| 45 | capsul* contract*.ti,ab,kf. | 2387 |
| 46 | (reperfus* adj3 (injur* or damag*)).ti,ab,kf. | 48699 |
| 47 | ((post-cardiac arrest* or postresuscitation or post-resuscitat* or post-heart-arrest*) adj3 syndrome*).ti,ab,kf. | 458 |
| 48 | primary graft dysfunct*.ti,ab,kf. | 1376 |
| 49 | (surg* adj3 (shock or collaps*)).ti,ab,kf. | 2317 |
| 50 | (short* adj (bowel* or gut* or intestin*) adj syndrome*).ti,ab,kf. | 4318 |
| 51 | slit-ventricle*.ti,ab,kf. | 287 |
| 52 | ((wound* or suture* or surgical-wound*) adj3 (dehiscence* or disrupt* or rupture* or separat* or seperat*)).ti,ab,kf. | 9313 |
| 53 | (vasopleg* or vaso-pleg*).ti,ab,kf. | 735 |
| 54 | (bone* adj3 (cement* or glue* or paste*)).ti,ab,kf. | 10287 |
| 55 | (((breast* or mammar*) adj3 (implant* or prosthes* or endoprosthes)).ti,ab,kf. or Breast Implants/) and (rupt* or fail* or leak*).ti,ab,kf. | 1496 |
| 56 | Keratitis/ or (diffus* adj2 lamellar* adj2 keratit*).ti,ab,kf. | 9466 |
| 57 | (gastric*-band* adj3 (erode* or erosion* or fail*)).ti,ab,kf. | 291 |
| 58 | (hypocalcemi* or hypocalcaemi* or hungry-bone*).ti,ab,kf. | 14305 |
| 59 | ((low-anterior-resection or LAR) adj3 syndrome*).ti,ab,kf. | 488 |
| 60 | (paravalvular adj (leak* or regurgitat*)).ti,ab,kf. | 3217 |
| 61 | (malignan*-glaucoma or aqueous-misdirection or ciliar*-block-glaucoma).ti,ab,kf. | 521 |
| 62 | (pseudo* adj3 glaucoma).ti,ab,kf. | 1354 |
| 63 | ((pneumonectom* or post-pneumonectom* or postpneumonectom*) adj3 syndrome*).ti,ab,kf. | 177 |
| 64 | ((endoprosthe* or endo-prosthe* or prosthe* or periprosthe* or peri-prosthe* or screw*) adj3 (dysfunction* or disfunction* or deteriorat* or dislocat* or endocarditis* or erod* or erosion* or fail* or fracture* or loosen* or thrombosis)).ti,ab,kf. | 20684 |
| 65 | ((retain* adj3 (appliance* or body or bodies or clip* or cotton* or drain* or gauze* or linen* or item* or instrument* or material* or needle* or object* or pad or pads or sponge* or swab* or textile* or tool* or towel*)) or textiloma* or gossypiboma*).ti,ab,kf. | 6463 |
| 66 | ((surg* or operat*) adj3 infect*).ti,ab,kf. | 39952 |
| 67 | (scar* adj3 endometriosis*).ti,ab,kf. | 293 |
| 68 | or/9-67 | 2401957 |
| 69 | Asian American Native Hawaiian and Pacific Islander/ | 30 |
| 70 | Asian/ | 9442 |
| 71 | Asian People/ or East Asian People/ or Southeast Asian People/ or South Asian People/ | 80139 |
| 72 | Asia/ or Asia, Southeastern/ or Borneo/ or Brunei/ or Cambodia/ or Indochina/ or Indonesia/ or Laos/ or Malaysia/ or Mekong Valley/ or Myanmar/ or Philippines/ or Singapore/ or Thailand/ or Timor-Leste/ or Vietnam/ or Asia, Eastern/ or China/ or Beijing/ or Hong Kong/ or Macau/ or Tibet/ or Japan/ or Tokyo/ or Korea/ or "Democratic People's Republic of Korea"/ or "Republic of Korea"/ or Mongolia/ or Taiwan/ | 672681 |
| 73 | (Ainu* or Asia* or Bai* or Bangladesh* or Bhote* or Bhutan* or Bornean* or Borneo* or Brunei* or Burma* or Burmese* or Cambodia* or Ceylon* or China* or Chinese* or Darussalam* or Dayak* or Dyak* or Filipin* or Hmong* or Hong Kong* or Hongkong* or Hu or Huren or Huzu or Hus or India* or Indo-chin* or Indonesia* or Iu-Mien* or Japan* or Kalmyk* or Kampuchea* or Kampudja* or Karen* or Kashmir* or Khmer* or Korea* or Lao* or Macanes* or Macanese* or Macao* or Macau* or Malaysia* or Maldiv* or Manchu* or Maylay* or Melanesia* or Mien* or Mongol* or Mongolia* or Myanma* or Nepal* or Pakistan* or Paracel* or Philippin* or Ryukyuan* or Singapor* or Sinhal* or South*-asia* or Spratly* or Srilanka* or Sri-Lanka* or Taiwan* or Tamil* or Thai* or Tibet* or Timor* or Turkic* or Viet* or Zhuang*).ti,ab,kf. | 1622735 |
| 74 | or/70-73 | 1767212 |
| 75 | exp United States/ | 1475402 |
| 76 | (america* or united states).ti,ab,kf. | 795538 |
| 77 | (Alabama* or Alaska* or Arizona* or Arkansa* or California* or Carolina* or Colorad* or Connecticut* or Dakota* or Delaware* or District of Columbia or Florida* or Floridian* or Georgia* or Hawaii* or Idaho* or Illinois* or Indiana* or Iowa* or Kansas or Kansan or Kentuck* or Louisiana* or Maine* or Maryland* or Massachusetts* or Michigan* or Minnesota* or Mississippi* or Missouri* or Montana* or Nebraska* or Nevada* or New England* or New Hampshir* or New Jersey* or New Mexic* or New York* or North Carolin* or Ohio* or Oklahom* or Oregon* or Pennsylvania* or Puerto Ric* or Rhode Island* or South Carolin* or Tennesse* or Texas* or Texan* or Utah* or Vermont* or Virginia* or Washington* or West Virginia* or Wisconsin* or Wyoming*).ti,ab,kf. | 526896 |
| 78 | (New York or Los Angeles or Chicago or Houston or Phoenix or Philadelphia or San Antonio or San Diego or Dallas or Austin or Jacksonville or San Jose or ((Fort or Ft) adj Worth) or Columbus or Charlotte or Indianapolis or San Francisco or Seattle or Denver or Oklahoma City or Nashville or El Paso or Washington or Las Vegas or Boston or Portland or Louisville or Memphis or Detroit or Baltimore or Milwaukee or Albuquerque or Tucson or Fresno or Sacramento or Mesa or Kansas City or Atlanta or Colorado Springs or Omaha or Raleigh or Virginia Beach or Long Beach or Miami or Oakland or Minneapolis or Tulsa or Bakersfield or Tampa or Wichita or Arlington or Aurora or New Orleans or Cleveland or Anaheim or Honolulu or Henderson or Stockton or Riverside or Lexington or Corpus Christi or Orlando or Irvine or Cincinnati or Santa Ana or Newark or ((Saint or St) adj Paul) or Pittsburgh or Greensboro or Lincoln or Durham or Plano or Anchorage or Jersey City or ((Saint or St) adj Louis) or Chandler or North Las Vegas or Chula Vista or Buffalo or Gilbert or Reno or Madison or ((Fort or Ft) adj Wayne) or Toledo or Lubbock or ((Saint or St) adj Petersburg) or Laredo or Irving or Chesapeake or Glendale or Winston-Salem or Scottsdale or Garland or Boise or Norfolk or (Port adj (Saint or St) adj Lucie) or Spokane or Richmond or Fremont or Huntsville or Tacoma or Baton Rouge or Santa Clarita or San Bernardino or Hialeah or Frisco or Modesto or Cape Coral or Fontana or Moreno Valley or Des Moines or Rochester or Fayetteville or Yonkers or McKinney or Worcester or Salt Lake City or Little Rock or Columbus or Augusta or Sioux Falls or Grand Prairie or Tallahassee or Amarillo or Oxnard or Peoria or Overland Park or Montgomery or Birmingham or Grand Rapids or Knoxville or Vancouver or Huntington Beach or Providence or Brownsville or Glendale or Akron or Tempe or Newport News or Chattanooga or Mobile or ((Fort or Ft) adj Lauderdale) or Cary or Shreveport or Ontario or Eugene or Aurora or Elk Grove or Salem or Santa Rosa or Clarksville or Rancho Cucamonga or Oceanside or Springfield or Pembroke Pines or Garden Grove or ((Fort or Ft) adj Collins) or Lancaster or Palmdale or Murfreesboro or Salinas or Corona or Killeen or Hayward or Paterson or Macon or Lakewood or Alexandria or Roseville or Surprise or Springfield or Charleston or Kansas City or Sunnyvale or Bellevue or Hollywood or Denton or Escondido or Joliet or Naperville or Bridgeport or Savannah or Mesquite or Pasadena or Rockford or Pomona or Jackson or Olathe or Gainesville or McAllen or Syracuse or Waco or Visalia or Thornton or Torrance or Fullerton or Columbia or Lakewood or New Haven or Hampton or Miramar or Victorville or Warren or West Valley City or Cedar Rapids or Stamford or Orange or Dayton or Midland or Kent or Elizabeth or Pasadena or Carrollton or Coral Springs or Sterling Heights or Fargo or Lewisville or Meridian or Norman or Palm Bay or Athens or Columbia or Abilene or Pearland or Santa Clara or Round Rock or Topeka or Allentown or Clovis or Simi Valley or College Station or Thousand Oaks or Vallejo or Concord or Rochester or Arvada or Lafayette or Independence or West Palm Beach or Hartford or Wilmington or Lakeland or Billings or Ann Arbor or Fairfield or Berkeley or Richardson or North Charleston or Cambridge or Broken Arrow or Clearwater or West Jordan or Evansville or League City or Antioch or Manchester or High Point or Waterbury or Westminster or Richmond or Carlsbad or Las Cruces or Murrieta or Lowell or Provo or Springfield or Elgin or Odessa or Lansing or Pompano Beach or Beaumont or Temecula or Gresham or Allen or Pueblo or Everett or South Fulton or Peoria or Nampa or Tuscaloosa or Miami Gardens or Santa Maria or Downey or Concord or Ventura or Costa Mesa or Sugar Land or Menifee or Tyler or Sparks or Greeley or Rio Rancho or Sandy Springs or Dearborn or Jurupa Valley or Edison or Spokane Valley or Hillsboro or Davie or Green Bay or Centennial or Buckeye or Boulder or Goodyear or El Monte or West Covina or Brockton or New Braunfels or El Cajon or Edinburg or Renton or Burbank or Inglewood or Rialto or Lee's Summit or Bend or Woodbridge or South Bend or Wichita Falls or ((Saint or St) adj George) or Fishers or Carmel or Vacaville or Quincy or Conroe or Chico or San Mateo or Lynn or Albany or Hesperia or New Bedford or Davenport or Daly City).ti,ab,kf. | 804699 |
| 79 | or/75-78 | 2765081 |
| 80 | 69 or (74 and 79) | 180957 |
| 81 | 8 and 68 and 80 | 71 |

**Embase (Elsevier Embase.com)
Sources: Embase, Embase Classic, MEDLINE.
Searched February 9, 2024.
300 records retrieved.**

| No. | Query | Results |
| --- | --- | --- |
| #90 | #8 AND #75 AND #89 | 300 |
| #89 | #78 OR #88 | 263805 |
| #88 | #82 AND #87 | 261353 |
| #87 | #83 OR #84 OR #85 OR #86 | 4094489 |
| #86 | 'new york':ti,ab,kw OR 'los angeles':ti,ab,kw OR chicago:ti,ab,kw OR houston:ti,ab,kw OR phoenix:ti,ab,kw OR philadelphia:ti,ab,kw OR 'san antonio':ti,ab,kw OR 'san diego':ti,ab,kw OR dallas:ti,ab,kw OR austin:ti,ab,kw OR jacksonville:ti,ab,kw OR 'san jose':ti,ab,kw OR (((fort OR ft) NEXT/1 worth):ti,ab,kw) OR charlotte:ti,ab,kw OR indianapolis:ti,ab,kw OR 'san francisco':ti,ab,kw OR seattle:ti,ab,kw OR denver:ti,ab,kw OR 'oklahoma city':ti,ab,kw OR nashville:ti,ab,kw OR 'el paso':ti,ab,kw OR washington:ti,ab,kw OR 'las vegas':ti,ab,kw OR boston:ti,ab,kw OR portland:ti,ab,kw OR louisville:ti,ab,kw OR memphis:ti,ab,kw OR detroit:ti,ab,kw OR baltimore:ti,ab,kw OR milwaukee:ti,ab,kw OR albuquerque:ti,ab,kw OR tucson:ti,ab,kw OR fresno:ti,ab,kw OR sacramento:ti,ab,kw OR mesa:ti,ab,kw OR atlanta:ti,ab,kw OR 'colorado springs':ti,ab,kw OR omaha:ti,ab,kw OR raleigh:ti,ab,kw OR 'virginia beach':ti,ab,kw OR 'long beach':ti,ab,kw OR miami:ti,ab,kw OR oakland:ti,ab,kw OR minneapolis:ti,ab,kw OR tulsa:ti,ab,kw OR bakersfield:ti,ab,kw OR tampa:ti,ab,kw OR wichita:ti,ab,kw OR arlington:ti,ab,kw OR 'new orleans':ti,ab,kw OR cleveland:ti,ab,kw OR anaheim:ti,ab,kw OR honolulu:ti,ab,kw OR henderson:ti,ab,kw OR stockton:ti,ab,kw OR riverside:ti,ab,kw OR lexington:ti,ab,kw OR 'corpus christi':ti,ab,kw OR orlando:ti,ab,kw OR irvine:ti,ab,kw OR cincinnati:ti,ab,kw OR 'santa ana':ti,ab,kw OR newark:ti,ab,kw OR (((saint OR st) NEXT/1 paul):ti,ab,kw) OR pittsburgh:ti,ab,kw OR greensboro:ti,ab,kw OR lincoln:ti,ab,kw OR durham:ti,ab,kw OR plano:ti,ab,kw OR anchorage:ti,ab,kw OR 'jersey city':ti,ab,kw OR (((saint OR st) NEXT/1 louis):ti,ab,kw) OR chandler:ti,ab,kw OR 'north las vegas':ti,ab,kw OR 'chula vista':ti,ab,kw OR buffalo:ti,ab,kw OR gilbert:ti,ab,kw OR reno:ti,ab,kw OR madison:ti,ab,kw OR (((fort OR ft) NEXT/1 wayne):ti,ab,kw) OR toledo:ti,ab,kw OR lubbock:ti,ab,kw OR (((saint OR st) NEXT/1 petersburg):ti,ab,kw) OR laredo:ti,ab,kw OR irving:ti,ab,kw OR chesapeake:ti,ab,kw OR 'winston salem':ti,ab,kw OR scottsdale:ti,ab,kw OR garland:ti,ab,kw OR boise:ti,ab,kw OR norfolk:ti,ab,kw OR ((port NEXT/1 (saint OR st) NEXT/1 lucie):ti,ab,kw) OR spokane:ti,ab,kw OR fremont:ti,ab,kw OR huntsville:ti,ab,kw OR tacoma:ti,ab,kw OR 'baton rouge':ti,ab,kw OR 'santa clarita':ti,ab,kw OR 'san bernardino':ti,ab,kw OR hialeah:ti,ab,kw OR frisco:ti,ab,kw OR modesto:ti,ab,kw OR 'cape coral':ti,ab,kw OR fontana:ti,ab,kw OR 'moreno valley':ti,ab,kw OR 'des moines':ti,ab,kw OR fayetteville:ti,ab,kw OR yonkers:ti,ab,kw OR mckinney:ti,ab,kw OR worcester:ti,ab,kw OR 'salt lake city':ti,ab,kw OR 'little rock':ti,ab,kw OR columbus:ti,ab,kw OR augusta:ti,ab,kw OR 'sioux falls':ti,ab,kw OR 'grand prairie':ti,ab,kw OR tallahassee:ti,ab,kw OR amarillo:ti,ab,kw OR oxnard:ti,ab,kw OR 'overland park':ti,ab,kw OR montgomery:ti,ab,kw OR birmingham:ti,ab,kw OR 'grand rapids':ti,ab,kw OR knoxville:ti,ab,kw OR vancouver:ti,ab,kw OR 'huntington beach':ti,ab,kw OR providence:ti,ab,kw OR brownsville:ti,ab,kw OR glendale:ti,ab,kw OR akron:ti,ab,kw OR tempe:ti,ab,kw OR 'newport news':ti,ab,kw OR chattanooga:ti,ab,kw OR mobile:ti,ab,kw OR (((fort OR ft) NEXT/1 lauderdale):ti,ab,kw) OR cary:ti,ab,kw OR shreveport:ti,ab,kw OR ontario:ti,ab,kw OR eugene:ti,ab,kw OR aurora:ti,ab,kw OR 'elk grove':ti,ab,kw OR salem:ti,ab,kw OR 'santa rosa':ti,ab,kw OR clarksville:ti,ab,kw OR 'rancho cucamonga':ti,ab,kw OR oceanside:ti,ab,kw OR 'pembroke pines':ti,ab,kw OR 'garden grove':ti,ab,kw OR (((fort OR ft) NEXT/1 collins):ti,ab,kw) OR lancaster:ti,ab,kw OR palmdale:ti,ab,kw OR murfreesboro:ti,ab,kw OR salinas:ti,ab,kw OR corona:ti,ab,kw OR killeen:ti,ab,kw OR hayward:ti,ab,kw OR paterson:ti,ab,kw OR macon:ti,ab,kw OR alexandria:ti,ab,kw OR roseville:ti,ab,kw OR surprise:ti,ab,kw OR charleston:ti,ab,kw OR 'kansas city':ti,ab,kw OR sunnyvale:ti,ab,kw OR bellevue:ti,ab,kw OR hollywood:ti,ab,kw OR denton:ti,ab,kw OR escondido:ti,ab,kw OR joliet:ti,ab,kw OR naperville:ti,ab,kw OR bridgeport:ti,ab,kw OR savannah:ti,ab,kw OR mesquite:ti,ab,kw OR rockford:ti,ab,kw OR pomona:ti,ab,kw OR jackson:ti,ab,kw OR olathe:ti,ab,kw OR gainesville:ti,ab,kw OR mcallen:ti,ab,kw OR syracuse:ti,ab,kw OR waco:ti,ab,kw OR visalia:ti,ab,kw OR thornton:ti,ab,kw OR torrance:ti,ab,kw OR fullerton:ti,ab,kw OR lakewood:ti,ab,kw OR 'new haven':ti,ab,kw OR hampton:ti,ab,kw OR miramar:ti,ab,kw OR victorville:ti,ab,kw OR warren:ti,ab,kw OR 'west valley city':ti,ab,kw OR 'cedar rapids':ti,ab,kw OR stamford:ti,ab,kw OR orange:ti,ab,kw OR dayton:ti,ab,kw OR midland:ti,ab,kw OR kent:ti,ab,kw OR elizabeth:ti,ab,kw OR pasadena:ti,ab,kw OR carrollton:ti,ab,kw OR 'coral springs':ti,ab,kw OR 'sterling heights':ti,ab,kw OR fargo:ti,ab,kw OR lewisville:ti,ab,kw OR meridian:ti,ab,kw OR norman:ti,ab,kw OR 'palm bay':ti,ab,kw OR athens:ti,ab,kw OR columbia:ti,ab,kw OR abilene:ti,ab,kw OR pearland:ti,ab,kw OR 'santa clara':ti,ab,kw OR 'round rock':ti,ab,kw OR topeka:ti,ab,kw OR allentown:ti,ab,kw OR clovis:ti,ab,kw OR 'simi valley':ti,ab,kw OR 'college station':ti,ab,kw OR 'thousand oaks':ti,ab,kw OR vallejo:ti,ab,kw OR rochester:ti,ab,kw OR arvada:ti,ab,kw OR lafayette:ti,ab,kw OR independence:ti,ab,kw OR 'west palm beach':ti,ab,kw OR hartford:ti,ab,kw OR wilmington:ti,ab,kw OR lakeland:ti,ab,kw OR billings:ti,ab,kw OR 'ann arbor':ti,ab,kw OR fairfield:ti,ab,kw OR berkeley:ti,ab,kw OR richardson:ti,ab,kw OR 'north charleston':ti,ab,kw OR cambridge:ti,ab,kw OR 'broken arrow':ti,ab,kw OR clearwater:ti,ab,kw OR 'west jordan':ti,ab,kw OR evansville:ti,ab,kw OR 'league city':ti,ab,kw OR antioch:ti,ab,kw OR manchester:ti,ab,kw OR 'high point':ti,ab,kw OR waterbury:ti,ab,kw OR westminster:ti,ab,kw OR richmond:ti,ab,kw OR carlsbad:ti,ab,kw OR 'las cruces':ti,ab,kw OR murrieta:ti,ab,kw OR lowell:ti,ab,kw OR provo:ti,ab,kw OR springfield:ti,ab,kw OR elgin:ti,ab,kw OR odessa:ti,ab,kw OR lansing:ti,ab,kw OR 'pompano beach':ti,ab,kw OR beaumont:ti,ab,kw OR temecula:ti,ab,kw OR gresham:ti,ab,kw OR allen:ti,ab,kw OR pueblo:ti,ab,kw OR everett:ti,ab,kw OR 'south fulton':ti,ab,kw OR peoria:ti,ab,kw OR nampa:ti,ab,kw OR tuscaloosa:ti,ab,kw OR 'miami gardens':ti,ab,kw OR 'santa maria':ti,ab,kw OR downey:ti,ab,kw OR concord:ti,ab,kw OR ventura:ti,ab,kw OR 'costa mesa':ti,ab,kw OR 'sugar land':ti,ab,kw OR menifee:ti,ab,kw OR tyler:ti,ab,kw OR sparks:ti,ab,kw OR greeley:ti,ab,kw OR 'rio rancho':ti,ab,kw OR 'sandy springs':ti,ab,kw OR dearborn:ti,ab,kw OR 'jurupa valley':ti,ab,kw OR edison:ti,ab,kw OR 'spokane valley':ti,ab,kw OR hillsboro:ti,ab,kw OR davie:ti,ab,kw OR 'green bay':ti,ab,kw OR centennial:ti,ab,kw OR buckeye:ti,ab,kw OR boulder:ti,ab,kw OR goodyear:ti,ab,kw OR 'el monte':ti,ab,kw OR 'west covina':ti,ab,kw OR brockton:ti,ab,kw OR 'new braunfels':ti,ab,kw OR 'el cajon':ti,ab,kw OR edinburg:ti,ab,kw OR renton:ti,ab,kw OR burbank:ti,ab,kw OR inglewood:ti,ab,kw OR rialto:ti,ab,kw OR 'lees summit':ti,ab,kw OR bend:ti,ab,kw OR woodbridge:ti,ab,kw OR 'south bend':ti,ab,kw OR 'wichita falls':ti,ab,kw OR (((saint OR st) NEXT/1 george):ti,ab,kw) OR fishers:ti,ab,kw OR carmel:ti,ab,kw OR vacaville:ti,ab,kw OR quincy:ti,ab,kw OR conroe:ti,ab,kw OR chico:ti,ab,kw OR 'san mateo':ti,ab,kw OR lynn:ti,ab,kw OR albany:ti,ab,kw OR hesperia:ti,ab,kw OR 'new bedford':ti,ab,kw OR davenport:ti,ab,kw OR 'daly city':ti,ab,kw | 1222151 |
| #85 | alabama*:ti,ab,kw OR alaska*:ti,ab,kw OR arizona*:ti,ab,kw OR arkansa*:ti,ab,kw OR california*:ti,ab,kw OR carolina*:ti,ab,kw OR colorad*:ti,ab,kw OR connecticut*:ti,ab,kw OR dakota*:ti,ab,kw OR delaware*:ti,ab,kw OR 'district of columbia':ti,ab,kw OR florida*:ti,ab,kw OR floridian*:ti,ab,kw OR georgia*:ti,ab,kw OR hawaii*:ti,ab,kw OR idaho*:ti,ab,kw OR illinois*:ti,ab,kw OR indiana*:ti,ab,kw OR iowa*:ti,ab,kw OR kansas:ti,ab,kw OR kansan:ti,ab,kw OR kentuck*:ti,ab,kw OR louisiana*:ti,ab,kw OR maine*:ti,ab,kw OR maryland*:ti,ab,kw OR massachusetts*:ti,ab,kw OR michigan*:ti,ab,kw OR minnesota*:ti,ab,kw OR mississippi*:ti,ab,kw OR missouri*:ti,ab,kw OR montana*:ti,ab,kw OR nebraska*:ti,ab,kw OR nevada*:ti,ab,kw OR 'new england*':ti,ab,kw OR 'new hampshir*':ti,ab,kw OR 'new jersey*':ti,ab,kw OR 'new mexic*':ti,ab,kw OR 'new york*':ti,ab,kw OR 'north carolin*':ti,ab,kw OR ohio*:ti,ab,kw OR oklahom*:ti,ab,kw OR oregon*:ti,ab,kw OR pennsylvania*:ti,ab,kw OR 'puerto ric*':ti,ab,kw OR 'rhode island*':ti,ab,kw OR 'south carolin*':ti,ab,kw OR tennesse*:ti,ab,kw OR texas*:ti,ab,kw OR texan*:ti,ab,kw OR utah*:ti,ab,kw OR vermont*:ti,ab,kw OR virginia*:ti,ab,kw OR washington*:ti,ab,kw OR 'west virginia*':ti,ab,kw OR wisconsin*:ti,ab,kw OR wyoming*:ti,ab,kw | 711815 |
| #84 | america*:ti,ab,kw OR 'united states':ti,ab,kw | 1752494 |
| #83 | 'united states'/exp OR 'american'/de | 1610208 |
| #82 | #79 OR #80 OR #81 | 2227017 |
| #81 | ainu*:ti,ab,kw OR asia*:ti,ab,kw OR bai*:ti,ab,kw OR bangladesh*:ti,ab,kw OR bhote*:ti,ab,kw OR bhutan*:ti,ab,kw OR bornean*:ti,ab,kw OR borneo*:ti,ab,kw OR brunei*:ti,ab,kw OR burma*:ti,ab,kw OR burmese*:ti,ab,kw OR cambodia*:ti,ab,kw OR ceylon*:ti,ab,kw OR china*:ti,ab,kw OR chinese*:ti,ab,kw OR darussalam*:ti,ab,kw OR dayak*:ti,ab,kw OR dyak*:ti,ab,kw OR filipin*:ti,ab,kw OR hmong*:ti,ab,kw OR 'hong kong*':ti,ab,kw OR hongkong*:ti,ab,kw OR hu:ti,ab,kw OR huren:ti,ab,kw OR huzu:ti,ab,kw OR hus:ti,ab,kw OR india*:ti,ab,kw OR 'indo chin*':ti,ab,kw OR indonesia*:ti,ab,kw OR 'iu mien*':ti,ab,kw OR japan*:ti,ab,kw OR kalmyk*:ti,ab,kw OR kampuchea*:ti,ab,kw OR kampudja*:ti,ab,kw OR karen*:ti,ab,kw OR kashmir*:ti,ab,kw OR khmer*:ti,ab,kw OR korea*:ti,ab,kw OR lao*:ti,ab,kw OR macanes*:ti,ab,kw OR macanese*:ti,ab,kw OR macao*:ti,ab,kw OR macau*:ti,ab,kw OR malaysia*:ti,ab,kw OR maldiv*:ti,ab,kw OR manchu*:ti,ab,kw OR maylay*:ti,ab,kw OR melanesia*:ti,ab,kw OR mien*:ti,ab,kw OR mongol*:ti,ab,kw OR mongolia*:ti,ab,kw OR myanma*:ti,ab,kw OR nepal*:ti,ab,kw OR pakistan*:ti,ab,kw OR paracel*:ti,ab,kw OR philippin*:ti,ab,kw OR ryukyuan*:ti,ab,kw OR singapor*:ti,ab,kw OR sinhal*:ti,ab,kw OR 'south* asia*':ti,ab,kw OR spratly*:ti,ab,kw OR srilanka*:ti,ab,kw OR 'sri lanka*':ti,ab,kw OR taiwan*:ti,ab,kw OR tamil*:ti,ab,kw OR thai*:ti,ab,kw OR tibet*:ti,ab,kw OR timor*:ti,ab,kw OR turkic*:ti,ab,kw OR viet*:ti,ab,kw OR zhuang*:ti,ab,kw | 2170311 |
| #80 | 'asian'/de OR 'east asian'/exp OR 'south asian'/exp OR 'southeast asian'/exp | 258157 |
| #79 | 'asian continental ancestry group'/de | 6541 |
| #78 | #76 OR #77 | 10709 |
| #77 | 'asian american, native hawaiian and pacific islander'/de | 51 |
| #76 | 'asian american'/de OR 'asian american, native hawaiian and pacific islander'/de | 10719 |
| #75 | #9 OR #10 OR #11 OR #12 OR #13 OR #14 OR #15 OR #16 OR #17 OR #18 OR #19 OR #20 OR #21 OR #22 OR #23 OR #24 OR #25 OR #26 OR #27 OR #28 OR #29 OR #30 OR #31 OR #32 OR #33 OR #34 OR #35 OR #36 OR #37 OR #38 OR #39 OR #40 OR #41 OR #42 OR #43 OR #44 OR #45 OR #46 OR #47 OR #48 OR #49 OR #50 OR #51 OR #52 OR #53 OR #54 OR #55 OR #56 OR #57 OR #58 OR #59 OR #60 OR #61 OR #62 OR #63 OR #64 OR #65 OR #66 OR #67 OR #68 OR #69 OR #70 OR #71 OR #72 OR #73 OR #74 | 4017649 |
| #74 | (scar* NEAR/3 endometriosis*):ti,ab,kw | 428 |
| #73 | ((surg* OR operat*) NEAR/3 infect*):ti,ab,kw | 58937 |
| #72 | ((retain* NEAR/3 (appliance* OR body OR bodies OR clip* OR cotton* OR drain* OR gauze* OR linen* OR item* OR instrument* OR material* OR needle* OR object* OR pad OR pads OR sponge* OR swab* OR textile* OR tool* OR towel*)):ti,ab,kw) OR textiloma*:ti,ab,kw OR gossypiboma*:ti,ab,kw | 8239 |
| #71 | ((endoprosthe* OR 'endo prosthe*' OR prosthe* OR periprosthe* OR 'peri prosthe*' OR screw*) NEAR/3 (dysfunction* OR disfunction* OR deteriorat* OR dislocat* OR endocarditis* OR erod* OR erosion* OR fail* OR fracture* OR loosen* OR thrombosis)):ti,ab,kw | 25469 |
| #70 | ((pneumonectom* OR 'post pneumonectom*' OR postpneumonectom*) NEAR/3 syndrome*):ti,ab,kw | 210 |
| #69 | (pseudo* NEAR/3 glaucoma):ti,ab,kw | 1628 |
| #68 | 'malignan* glaucoma':ti,ab,kw OR 'aqueous misdirection':ti,ab,kw OR 'ciliar* block glaucoma':ti,ab,kw | 668 |
| #67 | (paravalvular NEXT/1 (leak* OR regurgitat*)):ti,ab,kw | 5575 |
| #66 | (('low anterior resection' OR lar) NEAR/3 syndrome*):ti,ab,kw | 825 |
| #65 | hypocalcemi*:ti,ab,kw OR hypocalcaemi*:ti,ab,kw OR 'hungry bone*':ti,ab,kw | 22622 |
| #64 | ('gastric* band*' NEAR/3 (erode* OR erosion* OR fail*)):ti,ab,kw | 663 |
| #63 | (diffus* NEAR/2 lamellar* NEAR/2 keratit*):ti,ab,kw | 326 |
| #62 | 'keratitis'/exp | 45337 |
| #61 | ((((breast* OR mammar*) NEAR/3 (implant* OR prosthes* OR endoprosthes)):ti,ab,kw) OR 'breast implants'/de) AND (rupt*:ti,ab,kw OR fail*:ti,ab,kw OR leak*:ti,ab,kw) | 1884 |
| #60 | (bone* NEAR/3 (cement* OR glue* OR paste*)):ti,ab,kw | 12514 |
| #59 | vasopleg*:ti,ab,kw OR 'vaso pleg*':ti,ab,kw | 1298 |
| #58 | ((wound* OR suture* OR 'surgical wound*') NEAR/3 (dehiscence* OR disrupt* OR rupture* OR separat* OR seperat*)):ti,ab,kw | 13913 |
| #57 | 'slit ventricle*':ti,ab,kw | 391 |
| #56 | (short* NEXT/1 (bowel* OR gut* OR intestin*) NEXT/1 syndrome*):ti,ab,kw | 6963 |
| #55 | (surg* NEAR/3 (shock OR collaps*)):ti,ab,kw | 3003 |
| #54 | 'primary graft dysfunct*':ti,ab,kw | 2826 |
| #53 | (('post-cardiac arrest*' OR postresuscitation OR 'post resuscitat*' OR 'post heart arrest*') NEAR/3 syndrome*):ti,ab,kw | 757 |
| #52 | (reperfus* NEAR/3 (injur* OR damag*)):ti,ab,kw | 68344 |
| #51 | 'capsul* contract*':ti,ab,kw | 2689 |
| #50 | ((prosthesis* OR prosthetic*) NEAR/3 (fail* OR complicat* OR durab* OR loos* OR migrat* OR infect* OR surviv*)):ti,ab,kw | 18056 |
| #49 | 'prosthesis infection'/exp | 13971 |
| #48 | postcommissurotom*:ti,ab,kw OR postpericardiotom*:ti,ab,kw OR 'post commissurotom*':ti,ab,kw OR 'post pericardiotom*':ti,ab,kw | 718 |
| #47 | ponv:ti,ab,kw | 4375 |
| #46 | endoleak*:ti,ab,kw OR 'endo leak*':ti,ab,kw OR 'perigraft leak*':ti,ab,kw | 9445 |
| #45 | 'endoleak'/de | 10146 |
| #44 | postgastrectom*:ti,ab,kw OR 'post gastrectom*':ti,ab,kw OR 'stomach resect*':ti,ab,kw OR dumping*:ti,ab,kw | 9723 |
| #43 | cholecystectom*:ti,ab,kw OR 'post cholecystectom*':ti,ab,kw OR postcholecystectom*:ti,ab,kw OR choledochoduodenostom*:ti,ab,kw OR 'post choledochoduodenostom*':ti,ab,kw OR postcholedochoduodenostom*:ti,ab,kw OR sump:ti,ab,kw | 54944 |
| #42 | pseudomeli*:ti,ab,kw OR 'pseudo meli*':ti,ab,kw OR ((phantom NEAR/3 (limb* OR pain* OR sensation*)):ti,ab,kw) | 3931 |
| #41 | ((cicatr* OR incision* OR scar*) NEAR/3 hernia*):ti,ab,kw | 9970 |
| #40 | ((graft* OR allograft* OR transplant* OR flap*) NEAR/2 (occlusion* OR occlude* OR restenosi* OR disease* OR disorder* OR dysfunct* OR fail* OR infect* OR necros* OR reject* OR vasculopath*)):ti,ab,kw | 139989 |
| #39 | 'failed back surg*':ti,ab,kw OR ((('failed back' OR 'post laminectom*' OR postlaminectom*) NEAR/2 syndrome*):ti,ab,kw) OR fbss:ti,ab,kw | 2834 |
| #38 | ((emerg* OR 'post an$esth*' OR postan$esth*) NEXT/1 (deliri* OR agitat* OR excit*)):ti,ab,kw | 1470 |
| #37 | (residual NEAR/2 (block* OR curarizat*)):ti,ab,kw | 1924 |
| #36 | delay* AND ((emerg* NEXT/2 anesthes*):ti,ab,kw) | 169 |
| #35 | (delay* NEAR/2 (awake* OR conscious*)):ti,ab,kw | 359 |
| #34 | (delay* NEAR/2 (awake* OR conscious*)):ti,ab,kw | 359 |
| #33 | 'coronary-subclavian steal':ti,ab,kw | 306 |
| #32 | (cornea* NEXT/1 endothel* NEAR/2 (loss* OR damag* OR injur*)):ti,ab,kw | 904 |
| #31 | 'corneal endothelial cell loss'/de | 1118 |
| #30 | ((breast* NEXT/2 cancer NEAR/3 (lymphedem* OR lymphoedem*)):ti,ab,kw) OR ((mastectom* NEAR/3 (lymphedem* OR lymphoedem*)):ti,ab,kw) | 1912 |
| #29 | 'breast cancer-related lymphedema'/de | 1030 |
| #28 | ((anastomosis* OR anastomotic*) NEAR/3 (dehiscen* OR leak* OR rupture*)):ti,ab,kw | 23996 |
| #27 | ((afferent* OR efferent*) NEXT/1 (loop* OR limb*) NEAR/3 (syndrome* OR disease* OR obstruct*)):ti,ab,kw | 723 |
| #26 | 'surgery'/exp AND ('adverse device effect'/lnk OR 'adverse drug reaction'/lnk OR 'complication'/lnk OR 'drug interaction'/lnk OR 'drug toxicity'/lnk OR 'side effect'/lnk OR 'unexpected outcome of drug treatment'/lnk) | 1199324 |
| #25 | 'postoperative complication'/exp | 893142 |
| #24 | ((anesthesi* OR anesthaesi* OR malignan*) NEAR/4 (hypertherm* OR hyperpyrex*)):ti,ab,kw | 6322 |
| #23 | ((surg* OR operat*) NEAR/2 (advers* OR injur* OR complicat* OR 'blood loss' OR hemorrhag* OR haemorrhag*)):ti,ab,kw | 154196 |
| #22 | 'peroperative complication'/exp OR 'operative blood loss'/de OR 'intraoperative awareness'/de OR 'malignant hyperthermia'/de | 107161 |
| #21 | postanesthes*:ti,ab,kw OR 'post anesthes*':ti,ab,kw OR postanaesthes*:ti,ab,kw OR 'post anesthaes*':ti,ab,kw OR ((recover* NEXT/1 (room* OR ward* OR period*)):ti,ab,kw) OR pacu:ti,ab,kw | 34620 |
| #20 | 'anesthetic recovery'/de | 9195 |
| #19 | (block* NEAR/3 (autonomic OR cauda OR caudal OR cervical OR conduction OR dural OR epidural OR extradural OR ganglion OR intercostal OR lumbar OR nerve OR neurogenic OR neuromuscular OR paracervical OR peridural OR 'ql' OR 'quadratus lumborum' OR retrobulbar OR retroocular OR spinal OR 'stellate ganglion' OR subarachnoid OR tap OR 'transverse abdominal plane')):ti,ab,kw | 74712 |
| #18 | cryoanaesthe*:ti,ab,kw OR 'cryo anaesthe*':ti,ab,kw OR cryoanesthe*:ti,ab,kw OR 'cryo anesthe*':ti,ab,kw OR 'electro anaesthe*':ti,ab,kw OR electroanasthaesia:ti,ab,kw OR electroanesthe*:ti,ab,kw OR 'electro anesthe*':ti,ab,kw OR 'neuro anaesthe*':ti,ab,kw OR neuroanasthaesia:ti,ab,kw OR neuroanesthe*:ti,ab,kw OR 'neuro anesthe*':ti,ab,kw OR neuroleptanaesthe*:ti,ab,kw OR 'neuro leptanaesthe*':ti,ab,kw OR neuroleptanesthe*:ti,ab,kw OR 'neuro leptanesthe*':ti,ab,kw | 1756 |
| #17 | 'anesthesiological procedure'/exp | 1058530 |
| #16 | 'perioperative nursing'/exp | 7982 |
| #15 | periop*:ti,ab,kw OR 'peri op*':ti,ab,kw OR perisurg*:ti,ab,kw OR 'peri surg*':ti,ab,kw OR perop*:ti,ab,kw OR 'per op*':ti,ab,kw OR persurg*:ti,ab,kw OR 'per surg*':ti,ab,kw | 218772 |
| #14 | 'perioperative period'/de OR 'perioperative care'/de OR 'peroperative care'/de OR 'perioperative medicine'/de | 82980 |
| #13 | ((operat* OR surg* OR proced*) NEAR/7 sequelae*):ti,ab,kw | 5810 |
| #12 | postop*:ti,ab,kw OR 'post op*':ti,ab,kw OR postsurg*:ti,ab,kw OR 'post surg*':ti,ab,kw OR 'post proced*':ti,ab,kw | 1189843 |
| #11 | 'postoperative period'/exp | 671597 |
| #10 | ((oper* OR surg*) NEAR/2 (durat* OR length* OR time*)):ti,ab,kw | 232042 |
| #9 | intraop*:ti,ab,kw OR 'intra op*':ti,ab,kw OR intrasurg*:ti,ab,kw OR 'intra surg*':ti,ab,kw | 284450 |
| #8 | #1 OR #2 OR #3 OR #4 OR #5 OR #6 OR #7 | 650068 |
| #7 | glucosaemi*:ti,ab,kw OR glucosemi*:ti,ab,kw OR glycaemi*:ti,ab,kw OR glycemi*:ti,ab,kw OR hyperglycemi*:ti,ab,kw OR 'hyper glycemi*':ti,ab,kw OR hyperglycaemi*:ti,ab,kw OR 'hyper glycaemi*':ti,ab,kw OR hyperglucemi*:ti,ab,kw | 224563 |
| #6 | a1c:ti,ab,kw OR 'hb a1c':ti,ab,kw OR 'haemoglobin a1c':ti,ab,kw OR 'haemoglobin a 1c':ti,ab,kw OR 'haemoglobin aic':ti,ab,kw OR 'hba 1c':ti,ab,kw OR hba1c:ti,ab,kw OR 'hemoglobin a1c':ti,ab,kw OR 'hemoglobin a 1c':ti,ab,kw OR 'hemoglobin aic':ti,ab,kw | 128410 |
| #5 | ((metabolic* OR cardiometabolic OR 'cardio metabolic*' OR dysmetabolic* OR 'dys metabolic*' OR 'insulin resistan*' OR reaven*) NEAR/3 syndrom*):ti,ab,kw | 110868 |
| #4 | 'metabolic syndrome x'/de | 103507 |
| #3 | 'dm 2*':ti,ab,kw OR dm2*:ti,ab,kw OR mody*:ti,ab,kw OR niddm*:ti,ab,kw OR t2d*:ti,ab,kw OR t2dm*:ti,ab,kw | 109425 |
| #2 | (diabet* NEAR/2 ('adult onset' OR 'insulin independent*' OR 'ketosis resistant' OR latent* OR 'matur* onset' OR 'non insulin dependent' OR 'slow onset' OR stable OR 'type 2' OR 'type ii')):ti,ab,kw | 321095 |
| #1 | 'non insulin dependent diabetes mellitus'/de | 349877 |

**Cochrane Central Register of Controlled Trials: CENTRAL (Wiley)
Searched February 9, 2024.
59 records retrieved.**

| ID | Search |
| --- | --- |
| #1 | [mh "Diabetes Mellitus, Type 2"] |
| #2 | (Diabet*:ti,ab,kw NEAR/2 (adult-onset:ti,ab,kw OR (insulin independent*):ti,ab,kw OR (ketosis resistant):ti,ab,kw OR latent*:ti,ab,kw OR (matur* onset):ti,ab,kw OR non-insulin-dependent:ti,ab,kw OR slow-onset:ti,ab,kw OR stable:ti,ab,kw OR type-2:ti,ab,kw OR type-II:ti,ab,kw)) |
| #3 | (DM-2*:ti,ab,kw OR DM2*:ti,ab,kw OR MODY*:ti,ab,kw OR NIDDM*:ti,ab,kw OR T2D*:ti,ab,kw OR T2DM*:ti,ab,kw) |
| #4 | [mh ^"Metabolic Syndrome"] |
| #5 | ((metabolic*:ti,ab,kw OR cardiometabolic:ti,ab,kw OR (cardio metabolic*):ti,ab,kw OR dysmetabolic*:ti,ab,kw OR (dys-metabolic*):ti,ab,kw OR (insulin resistan*):ti,ab,kw OR reaven*:ti,ab,kw) NEAR/3 syndrom*:ti,ab,kw) |
| #6 | (a1c:ti,ab,kw OR hb-a1c:ti,ab,kw OR haemoglobin-a1c:ti,ab,kw OR haemoglobin-a-1c:ti,ab,kw OR haemoglobin-aic:ti,ab,kw OR hba-1c:ti,ab,kw OR hba1c:ti,ab,kw OR hemoglobin-a1c:ti,ab,kw OR hemoglobin-a-1c:ti,ab,kw OR hemoglobin-aic:ti,ab,kw) |
| #7 | (glucosaemi*:ti,ab,kw OR glucosemi*:ti,ab,kw OR glycaemi*:ti,ab,kw OR glycemi*:ti,ab,kw OR hyperglycaemi*:ti,ab,kw OR hyperglycemia*:ti,ab,kw OR hyper-glycemia*:ti,ab,kw OR hyperglycaemi*:ti,ab,kw OR hyper-glycaemi*:ti,ab,kw OR hyperglucemi*:ti,ab,kw) |
| #8 | {OR #1-#7} |
| #9 | [mh ^"Postoperative Period"] OR [mh ^"Postoperative Care"] |
| #10 | [mh "Perioperative Care"] OR [mh "Perioperative Period"] OR [mh ^"Perioperative Medicine"] |
| #11 | [mh "Perioperative Nursing"] |
| #12 | [mh ^"Intraoperative Complications"] OR [mh ^"Blood Loss, Surgical"] OR [mh ^"Intraoperative Awareness"] OR [mh ^"Malignant Hyperthermia"] |
| #13 | [mh "Postoperative Complications"] |
| #14 | [mh "Surgical Procedures, Operative"/ae] |
| #15 | [mh "Surgical Procedures, Operative"/co] |
| #16 | [mh ^"Graft Rejection"] |
| #17 | [mh Anesthesia] OR [mh "Conscious Sedation"] OR [mh "Deep Sedation"] OR [mh "Neuromuscular Blockade"] OR [mh "Preanesthetic Medication"] |
| #18 | (Intraop*:ti,ab,kw OR Intra-op*:ti,ab,kw OR intrasurg*:ti,ab,kw OR intra-surg*:ti,ab,kw) |
| #19 | ((oper*:ti,ab,kw OR surg*:ti,ab,kw) NEAR/2 (durat*:ti,ab,kw OR length*:ti,ab,kw OR time*:ti,ab,kw)) |
| #20 | (postop*:ti,ab,kw OR post-op*:ti,ab,kw OR postsurg*:ti,ab,kw OR post-surg*:ti,ab,kw OR post-proced*:ti,ab,kw) |
| #21 | ((operat*:ti,ab,kw OR surg*:ti,ab,kw OR proced*:ti,ab,kw) NEAR/7 sequelae*:ti,ab,kw) |
| #22 | (periop*:ti,ab,kw OR peri-op*:ti,ab,kw OR perisurg*:ti,ab,kw OR peri-surg*:ti,ab,kw) |
| #23 | (postanesthes*:ti,ab,kw OR post-anesthes*:ti,ab,kw OR postanaesthes*:ti,ab,kw OR post-anesthaes*:ti,ab,kw OR (recover*:ti,ab,kw NEXT (room*:ti,ab,kw OR ward*:ti,ab,kw OR period*:ti,ab,kw)) OR PACU:ti,ab,kw) |
| #24 | ((surg*:ti,ab,kw OR operat*:ti,ab,kw) NEAR/2 (advers*:ti,ab,kw OR injur*:ti,ab,kw OR complicat*:ti,ab,kw OR blood-loss:ti,ab,kw OR hemorrhag*:ti,ab,kw OR haemorrhag*:ti,ab,kw)) |
| #25 | ((anesthesi*:ti,ab,kw OR anesthaesi*:ti,ab,kw OR malignan*:ti,ab,kw) NEAR/4 (hypertherm*:ti,ab,kw OR hyperpyrex*:ti,ab,kw)) |
| #26 | ((afferent*:ti,ab,kw OR efferent*:ti,ab,kw) NEXT (loop*:ti,ab,kw OR limb*:ti,ab,kw) NEAR/3 (syndrome*:ti,ab,kw OR disease*:ti,ab,kw OR obstruct*:ti,ab,kw)) |
| #27 | ((anastomosis*:ti,ab,kw OR anastomotic*:ti,ab,kw) NEAR/3 (dehiscen*:ti,ab,kw OR leak*:ti,ab,kw OR rupture*:ti,ab,kw)) |
| #28 | (((breast*:ti,ab,kw NEAR/2 cancer:ti,ab,kw) OR mastectom*:ti,ab,kw) NEAR/3 (lymphedem*:ti,ab,kw OR lymphoedem*:ti,ab,kw)) |
| #29 | (cornea*:ti,ab,kw NEXT endothel*:ti,ab,kw NEAR/2 (loss*:ti,ab,kw OR damag*:ti,ab,kw OR injur*:ti,ab,kw)) |
| #30 | coronary-subclavian steal:ti,ab,kw |
| #31 | ((delay*:ti,ab,kw NEAR/2 (awake*:ti,ab,kw OR conscious*:ti,ab,kw OR (emergenc*:ti,ab,kw NEAR/2 anesth?es*:ti,ab,kw))) OR (residual:ti,ab,kw NEAR/2 (block*:ti,ab,kw OR curarizat*:ti,ab,kw))) |
| #32 | ((emerg*:ti,ab,kw OR post-an?esth*:ti,ab,kw OR postan?esth*:ti,ab,kw) NEXT (deliri*:ti,ab,kw OR agitat*:ti,ab,kw OR excit*:ti,ab,kw)) |
| #33 | (("failed back" NEXT surg*):ti,ab,kw OR ((failed-back:ti,ab,kw OR post-laminectom*:ti,ab,kw OR postlaminectom*:ti,ab,kw) NEAR/2 syndrome*:ti,ab,kw) OR FBSS:ti,ab,kw) |
| #34 | ((graft*:ti,ab,kw OR allograft*:ti,ab,kw OR transplant*:ti,ab,kw OR flap*:ti,ab,kw) NEAR/2 (occlusion*:ti,ab,kw OR occlude*:ti,ab,kw OR restenosi*:ti,ab,kw OR disease*:ti,ab,kw OR disorder*:ti,ab,kw OR dysfunct*:ti,ab,kw OR fail*:ti,ab,kw OR infect*:ti,ab,kw OR necros*:ti,ab,kw OR reject*:ti,ab,kw OR vasculopath*:ti,ab,kw)) |
| #35 | ((cicatr*:ti,ab,kw OR incision*:ti,ab,kw OR scar*:ti,ab,kw) NEAR/3 hernia*:ti,ab,kw) |
| #36 | (pseudomeli*:ti,ab,kw OR pseudo-meli*:ti,ab,kw OR (phantom:ti,ab,kw NEAR/3 (limb*:ti,ab,kw OR pain*:ti,ab,kw OR sensation*:ti,ab,kw))) |
| #37 | (cholecystectom*:ti,ab,kw OR post-cholecystectom*:ti,ab,kw OR postcholecystectom*:ti,ab,kw OR choledochoduodenostom*:ti,ab,kw OR post-choledochoduodenostom*:ti,ab,kw OR postcholedochoduodenostom*:ti,ab,kw OR sump:ti,ab,kw) |
| #38 | (postgastrectom*:ti,ab,kw OR post-gastrectom*:ti,ab,kw OR stomach-resect*:ti,ab,kw OR dumping*:ti,ab,kw) |
| #39 | (endoleak*:ti,ab,kw OR endo-leak*:ti,ab,kw OR perigraft-leak*:ti,ab,kw) |
| #40 | ponv:ti,ab,kw |
| #41 | (postcommissurotom*:ti,ab,kw OR postpericardiotom*:ti,ab,kw OR post-commissurotom*:ti,ab,kw OR post-pericardiotom*:ti,ab,kw) |
| #42 | ((prosthesis*:ti,ab,kw OR prosthetic*:ti,ab,kw) NEAR/3 (fail*:ti,ab,kw OR complicat*:ti,ab,kw OR durab*:ti,ab,kw OR loos*:ti,ab,kw OR migrat*:ti,ab,kw OR infect*:ti,ab,kw OR surviv*:ti,ab,kw)) |
| #43 | (capsul* NEXT contract*):ti,ab,kw |
| #44 | (reperfus*:ti,ab,kw NEAR/3 (injur*:ti,ab,kw OR damag*:ti,ab,kw)) |
| #45 | ((("post-cardiac" NEXT arrest*):ti,ab,kw OR postresuscitation:ti,ab,kw OR post-resuscitat*:ti,ab,kw OR post-heart-arrest*:ti,ab,kw) NEAR/3 syndrome*:ti,ab,kw) |
| #46 | ("primary graft" NEXT dysfunct*):ti,ab,kw |
| #47 | (surg*:ti,ab,kw NEAR/3 (shock:ti,ab,kw OR collaps*:ti,ab,kw)) |
| #48 | (short*:ti,ab,kw NEXT (bowel*:ti,ab,kw OR gut*:ti,ab,kw OR intestin*:ti,ab,kw) NEXT syndrome*:ti,ab,kw) |
| #49 | slit-ventricle*:ti,ab,kw |
| #50 | ((wound*:ti,ab,kw OR suture*:ti,ab,kw OR surgical-wound*:ti,ab,kw) NEAR/3 (dehiscence*:ti,ab,kw OR disrupt*:ti,ab,kw OR rupture*:ti,ab,kw OR separat*:ti,ab,kw OR seperat*:ti,ab,kw)) |
| #51 | (vasopleg*:ti,ab,kw OR vaso-pleg*:ti,ab,kw) |
| #52 | (bone*:ti,ab,kw NEAR/3 (cement*:ti,ab,kw OR glue*:ti,ab,kw OR paste*:ti,ab,kw)) |
| #53 | (((breast*:ti,ab,kw OR mammar*:ti,ab,kw) NEAR/3 (implant*:ti,ab,kw OR prosthes*:ti,ab,kw OR endoprosthes:ti,ab,kw)) OR [mh ^"Breast Implants"]) AND (rupt*:ti,ab,kw OR fail*:ti,ab,kw OR leak*:ti,ab,kw) |
| #54 | [mh ^Keratitis] OR (diffus*:ti,ab,kw NEAR/2 lamellar*:ti,ab,kw NEAR/2 keratit*:ti,ab,kw) |
| #55 | (gastric* NEXT/1 band*:ti,ab,kw NEAR/3 (erode*:ti,ab,kw OR erosion*:ti,ab,kw OR fail*:ti,ab,kw)) |
| #56 | (hypocalcemi*:ti,ab,kw OR hypocalcaemi*:ti,ab,kw OR hungry-bone*:ti,ab,kw) |
| #57 | ((low-anterior-resection:ti,ab,kw OR LAR:ti,ab,kw) NEAR/3 syndrome*:ti,ab,kw) |
| #58 | (paravalvular:ti,ab,kw NEXT (leak*:ti,ab,kw OR regurgitat*:ti,ab,kw)) |
| #59 | (malignan* NEXT/1 glaucoma:ti,ab,kw OR aqueous-misdirection:ti,ab,kw OR ciliar* NEXT/1 block-glaucoma:ti,ab,kw) |
| #60 | (pseudo*:ti,ab,kw NEAR/3 glaucoma:ti,ab,kw) |
| #61 | ((pneumonectom*:ti,ab,kw OR post-pneumonectom*:ti,ab,kw OR postpneumonectom*:ti,ab,kw) NEAR/3 syndrome*:ti,ab,kw) |
| #62 | ((endoprosthe*:ti,ab,kw OR endo-prosthe*:ti,ab,kw OR prosthe*:ti,ab,kw OR periprosthe*:ti,ab,kw OR peri-prosthe*:ti,ab,kw OR screw*:ti,ab,kw) NEAR/3 (dysfunction*:ti,ab,kw OR disfunction*:ti,ab,kw OR deteriorat*:ti,ab,kw OR dislocat*:ti,ab,kw OR endocarditis*:ti,ab,kw OR erod*:ti,ab,kw OR erosion*:ti,ab,kw OR fail*:ti,ab,kw OR fracture*:ti,ab,kw OR loosen*:ti,ab,kw OR thrombosis:ti,ab,kw)) |
| #63 | ((retain*:ti,ab,kw NEAR/3 (appliance*:ti,ab,kw OR body:ti,ab,kw OR bodies:ti,ab,kw OR clip*:ti,ab,kw OR cotton*:ti,ab,kw OR drain*:ti,ab,kw OR gauze*:ti,ab,kw OR linen*:ti,ab,kw OR item*:ti,ab,kw OR instrument*:ti,ab,kw OR material*:ti,ab,kw OR needle*:ti,ab,kw OR object*:ti,ab,kw OR pad:ti,ab,kw OR pads:ti,ab,kw OR sponge*:ti,ab,kw OR swab*:ti,ab,kw OR textile*:ti,ab,kw OR tool*:ti,ab,kw OR towel*:ti,ab,kw)) OR textiloma*:ti,ab,kw OR gossypiboma*:ti,ab,kw) |
| #64 | ((surg*:ti,ab,kw OR operat*:ti,ab,kw) NEAR/3 infect*:ti,ab,kw) |
| #65 | (scar*:ti,ab,kw NEAR/3 endometriosis*:ti,ab,kw) |
| #66 | (anesthe*:ti,ab,kw OR anaesthe*:ti,ab,kw OR cryoanaesthe*:ti,ab,kw OR cryo-anaesthe*:ti,ab,kw OR cryoanesthe*:ti,ab,kw OR cryo-anesthe*:ti,ab,kw OR electro-anaesthe*:ti,ab,kw OR electroanasthaesia:ti,ab,kw OR electroanesthe*:ti,ab,kw OR electro-anesthe*:ti,ab,kw OR neuro-anaesthe*:ti,ab,kw OR neuroanasthaesia:ti,ab,kw OR neuroanesthe*:ti,ab,kw OR neuro-anesthe*:ti,ab,kw OR neuroleptanaesthe*:ti,ab,kw OR neuro-leptanaesthe*:ti,ab,kw OR neuroleptanesthe*:ti,ab,kw OR neuro-leptanesthe*:ti,ab,kw) |
| #67 | (block*:ti,ab,kw NEAR/3 (autonomic:ti,ab,kw OR cauda:ti,ab,kw OR caudal:ti,ab,kw OR cervical:ti,ab,kw OR conduction:ti,ab,kw OR dural:ti,ab,kw OR epidural:ti,ab,kw OR extradural:ti,ab,kw OR ganglion:ti,ab,kw OR intercostal:ti,ab,kw OR lumbar:ti,ab,kw OR nerve:ti,ab,kw OR neurogenic:ti,ab,kw OR neuromuscular:ti,ab,kw OR paracervical:ti,ab,kw OR peridural:ti,ab,kw OR "'QL '":ti,ab,kw OR "quadratus lumborum":ti,ab,kw OR retrobulbar:ti,ab,kw OR retroocular:ti,ab,kw OR spinal:ti,ab,kw OR stellate-ganglion:ti,ab,kw OR subarachnoid:ti,ab,kw OR TAP:ti,ab,kw OR "transverse abdominal plane":ti,ab,kw)) |
| #68 | {OR #9-#67} |
| #69 | [mh "Asian American Native Hawaiian and Pacific Islander"] |
| #70 | [mh ^Asian] OR [mh ^"Asian People"] OR [mh ^"East Asian People"] OR [mh ^"Southeast Asian People"] OR [mh ^"South Asian People"] |
| #71 | [mh ^Asia] OR [mh ^"Asia, Southeastern"] OR [mh ^Borneo] OR [mh ^Brunei] OR [mh ^Cambodia] OR [mh ^Indochina] OR [mh ^Indonesia] OR [mh ^Laos] OR [mh ^Malaysia] OR [mh ^"Mekong Valley"] OR [mh ^Myanmar] OR [mh ^Philippines] OR [mh ^Singapore] OR [mh ^Thailand] OR [mh ^Vietnam] OR [mh ^"Asia, Eastern"] OR [mh ^China] OR [mh ^Beijing] OR [mh ^"Hong Kong"] OR [mh ^Macau] OR [mh ^Tibet] OR [mh ^Japan] OR [mh ^Tokyo] OR [mh ^Korea] OR [mh ^"Democratic People's Republic of Korea"] OR [mh ^"Republic of Korea"] OR [mh ^Mongolia] OR [mh ^Taiwan] |
| #72 | [mh ^Timor-Leste] |
| #73 | (Ainu*:ti,ab,kw OR Asia*:ti,ab,kw OR Bai*:ti,ab,kw OR Bangladesh*:ti,ab,kw OR Bhote*:ti,ab,kw OR Bhutan*:ti,ab,kw OR Bornean*:ti,ab,kw OR Borneo*:ti,ab,kw OR Brunei*:ti,ab,kw OR Burma*:ti,ab,kw OR Burmese*:ti,ab,kw OR Cambodia*:ti,ab,kw OR Ceylon*:ti,ab,kw OR China*:ti,ab,kw OR Chinese*:ti,ab,kw OR Darussalam*:ti,ab,kw OR Dayak*:ti,ab,kw OR Dyak*:ti,ab,kw OR Filipin*:ti,ab,kw OR Hmong*:ti,ab,kw OR ("Hong" NEXT Kong*):ti,ab,kw OR Hongkong*:ti,ab,kw OR Hu:ti,ab,kw OR Huren:ti,ab,kw OR Huzu:ti,ab,kw OR Hus:ti,ab,kw OR India*:ti,ab,kw OR (Indo NEXT/1 chin*:ti,ab,kw) OR Indonesia*:ti,ab,kw OR (Iu NEXT/1 Mien*:ti,ab,kw) OR Japan*:ti,ab,kw OR Kalmyk*:ti,ab,kw OR Kampuchea*:ti,ab,kw OR Kampudja*:ti,ab,kw OR Karen*:ti,ab,kw OR Kashmir*:ti,ab,kw OR Khmer*:ti,ab,kw OR Korea*:ti,ab,kw OR Lao*:ti,ab,kw OR Macanes*:ti,ab,kw OR Macanese*:ti,ab,kw OR Macao*:ti,ab,kw OR Macau*:ti,ab,kw OR Malaysia*:ti,ab,kw OR Maldiv*:ti,ab,kw OR Manchu*:ti,ab,kw OR Maylay*:ti,ab,kw OR Melanesia*:ti,ab,kw OR Mien*:ti,ab,kw OR Mongol*:ti,ab,kw OR Mongolia*:ti,ab,kw OR Myanma*:ti,ab,kw OR Nepal*:ti,ab,kw OR Pakistan*:ti,ab,kw OR Paracel*:ti,ab,kw OR Philippin*:ti,ab,kw OR Ryukyuan*:ti,ab,kw OR Singapor*:ti,ab,kw OR Sinhal*:ti,ab,kw OR (South* NEXT/1 Asia*:ti,ab,kw) OR Spratly*:ti,ab,kw OR Srilanka*:ti,ab,kw OR (Sri NEXT/1 Lanka*:ti,ab,kw) OR Taiwan*:ti,ab,kw OR Tamil*:ti,ab,kw OR Thai*:ti,ab,kw OR Tibet*:ti,ab,kw OR Timor*:ti,ab,kw OR Turkic*:ti,ab,kw OR Viet*:ti,ab,kw OR Zhuang*:ti,ab,kw) |
| #74 | {OR #69-#73} |
| #75 | [mh "United States"] |
| #76 | (america*:ti,ab,kw OR "united states":ti,ab,kw) |
| #77 | (Alabama*:ti,ab,kw OR Alaska*:ti,ab,kw OR Arizona*:ti,ab,kw OR Arkansa*:ti,ab,kw OR California*:ti,ab,kw OR Carolina*:ti,ab,kw OR Colorad*:ti,ab,kw OR Connecticut*:ti,ab,kw OR Dakota*:ti,ab,kw OR Delaware*:ti,ab,kw OR "District of Columbia":ti,ab,kw OR Florida*:ti,ab,kw OR Floridian*:ti,ab,kw OR Georgia*:ti,ab,kw OR Hawaii*:ti,ab,kw OR Idaho*:ti,ab,kw OR Illinois*:ti,ab,kw OR Indiana*:ti,ab,kw OR Iowa*:ti,ab,kw OR Kansas:ti,ab,kw OR Kansan:ti,ab,kw OR Kentuck*:ti,ab,kw OR Louisiana*:ti,ab,kw OR Maine*:ti,ab,kw OR Maryland*:ti,ab,kw OR Massachusetts*:ti,ab,kw OR Michigan*:ti,ab,kw OR Minnesota*:ti,ab,kw OR Mississippi*:ti,ab,kw OR Missouri*:ti,ab,kw OR Montana*:ti,ab,kw OR Nebraska*:ti,ab,kw OR Nevada*:ti,ab,kw OR ("New" NEXT England*):ti,ab,kw OR ("New" NEXT Hampshir*):ti,ab,kw OR ("New" NEXT Jersey*):ti,ab,kw OR ("New" NEXT Mexic*):ti,ab,kw OR ("New" NEXT York*):ti,ab,kw OR ("North" NEXT Carolin*):ti,ab,kw OR Ohio*:ti,ab,kw OR Oklahom*:ti,ab,kw OR Oregon*:ti,ab,kw OR Pennsylvania*:ti,ab,kw OR ("Puerto" NEXT Ric*):ti,ab,kw OR ("Rhode" NEXT Island*):ti,ab,kw OR ("South" NEXT Carolin*):ti,ab,kw OR Tennesse*:ti,ab,kw OR Texas*:ti,ab,kw OR Texan*:ti,ab,kw OR Utah*:ti,ab,kw OR Vermont*:ti,ab,kw OR Virginia*:ti,ab,kw OR Washington*:ti,ab,kw OR ("West" NEXT Virginia*):ti,ab,kw OR Wisconsin*:ti,ab,kw OR Wyoming*:ti,ab,kw) |
| #78 | ("New York":ti,ab,kw OR "Los Angeles":ti,ab,kw OR Chicago:ti,ab,kw OR Houston:ti,ab,kw OR Phoenix:ti,ab,kw OR Philadelphia:ti,ab,kw OR "San Antonio":ti,ab,kw OR "San Diego":ti,ab,kw OR Dallas:ti,ab,kw OR Austin:ti,ab,kw OR Jacksonville:ti,ab,kw OR "San Jose":ti,ab,kw OR ((Fort:ti,ab,kw OR Ft:ti,ab,kw) NEXT Worth:ti,ab,kw) OR Columbus:ti,ab,kw OR Charlotte:ti,ab,kw OR Indianapolis:ti,ab,kw OR "San Francisco":ti,ab,kw OR Seattle:ti,ab,kw OR Denver:ti,ab,kw OR "Oklahoma City":ti,ab,kw OR Nashville:ti,ab,kw OR "El Paso":ti,ab,kw OR Washington:ti,ab,kw OR "Las Vegas":ti,ab,kw OR Boston:ti,ab,kw OR Portland:ti,ab,kw OR Louisville:ti,ab,kw OR Memphis:ti,ab,kw OR Detroit:ti,ab,kw OR Baltimore:ti,ab,kw OR Milwaukee:ti,ab,kw OR Albuquerque:ti,ab,kw OR Tucson:ti,ab,kw OR Fresno:ti,ab,kw OR Sacramento:ti,ab,kw OR Mesa:ti,ab,kw OR "Kansas City":ti,ab,kw OR Atlanta:ti,ab,kw OR "Colorado Springs":ti,ab,kw OR Omaha:ti,ab,kw OR Raleigh:ti,ab,kw OR "Virginia Beach":ti,ab,kw OR "Long Beach":ti,ab,kw OR Miami:ti,ab,kw OR Oakland:ti,ab,kw OR Minneapolis:ti,ab,kw OR Tulsa:ti,ab,kw OR Bakersfield:ti,ab,kw OR Tampa:ti,ab,kw OR Wichita:ti,ab,kw OR Arlington:ti,ab,kw OR Aurora:ti,ab,kw OR "New Orleans":ti,ab,kw OR Cleveland:ti,ab,kw OR Anaheim:ti,ab,kw OR Honolulu:ti,ab,kw OR Henderson:ti,ab,kw OR Stockton:ti,ab,kw OR Riverside:ti,ab,kw OR Lexington:ti,ab,kw OR "Corpus Christi":ti,ab,kw OR Orlando:ti,ab,kw OR Irvine:ti,ab,kw OR Cincinnati:ti,ab,kw OR "Santa Ana":ti,ab,kw OR Newark:ti,ab,kw OR ((Saint:ti,ab,kw OR St:ti,ab,kw) NEXT Paul:ti,ab,kw) OR Pittsburgh:ti,ab,kw OR Greensboro:ti,ab,kw OR Lincoln:ti,ab,kw OR Durham:ti,ab,kw OR Plano:ti,ab,kw OR Anchorage:ti,ab,kw OR "Jersey City":ti,ab,kw OR ((Saint:ti,ab,kw OR St:ti,ab,kw) NEXT Louis:ti,ab,kw) OR Chandler:ti,ab,kw OR "North Las Vegas":ti,ab,kw OR "Chula Vista":ti,ab,kw OR Buffalo:ti,ab,kw OR Gilbert:ti,ab,kw OR Reno:ti,ab,kw OR Madison:ti,ab,kw OR ((Fort:ti,ab,kw OR Ft:ti,ab,kw) NEXT Wayne:ti,ab,kw) OR Toledo:ti,ab,kw OR Lubbock:ti,ab,kw OR ((Saint:ti,ab,kw OR St:ti,ab,kw) NEXT Petersburg:ti,ab,kw) OR Laredo:ti,ab,kw OR Irving:ti,ab,kw OR Chesapeake:ti,ab,kw OR Glendale:ti,ab,kw OR Winston-Salem:ti,ab,kw OR Scottsdale:ti,ab,kw OR Garland:ti,ab,kw OR Boise:ti,ab,kw OR Norfolk:ti,ab,kw OR (Port:ti,ab,kw NEXT (Saint:ti,ab,kw OR St:ti,ab,kw) NEXT Lucie:ti,ab,kw) OR Spokane:ti,ab,kw OR Richmond:ti,ab,kw OR Fremont:ti,ab,kw OR Huntsville:ti,ab,kw OR Tacoma:ti,ab,kw OR "Baton Rouge":ti,ab,kw OR "Santa Clarita":ti,ab,kw OR "San Bernardino":ti,ab,kw OR Hialeah:ti,ab,kw OR Frisco:ti,ab,kw OR Modesto:ti,ab,kw OR "Cape Coral":ti,ab,kw OR Fontana:ti,ab,kw OR "Moreno Valley":ti,ab,kw OR "Des Moines":ti,ab,kw OR Rochester:ti,ab,kw OR Fayetteville:ti,ab,kw OR Yonkers:ti,ab,kw OR McKinney:ti,ab,kw OR Worcester:ti,ab,kw OR "Salt Lake City":ti,ab,kw OR "Little Rock":ti,ab,kw OR Columbus:ti,ab,kw OR Augusta:ti,ab,kw OR "Sioux Falls":ti,ab,kw OR "Grand Prairie":ti,ab,kw OR Tallahassee:ti,ab,kw OR Amarillo:ti,ab,kw OR Oxnard:ti,ab,kw OR Peoria:ti,ab,kw OR "Overland Park":ti,ab,kw OR Montgomery:ti,ab,kw OR Birmingham:ti,ab,kw OR "Grand Rapids":ti,ab,kw OR Knoxville:ti,ab,kw OR Vancouver:ti,ab,kw OR "Huntington Beach":ti,ab,kw OR Providence:ti,ab,kw OR Brownsville:ti,ab,kw OR Glendale:ti,ab,kw OR Akron:ti,ab,kw OR Tempe:ti,ab,kw OR "Newport News":ti,ab,kw OR Chattanooga:ti,ab,kw OR Mobile:ti,ab,kw OR ((Fort:ti,ab,kw OR Ft:ti,ab,kw) NEXT Lauderdale:ti,ab,kw) OR Cary:ti,ab,kw OR Shreveport:ti,ab,kw OR Ontario:ti,ab,kw OR Eugene:ti,ab,kw OR Aurora:ti,ab,kw OR "Elk Grove":ti,ab,kw OR Salem:ti,ab,kw OR "Santa Rosa":ti,ab,kw OR Clarksville:ti,ab,kw OR "Rancho Cucamonga":ti,ab,kw OR Oceanside:ti,ab,kw OR Springfield:ti,ab,kw OR "Pembroke Pines":ti,ab,kw OR "Garden Grove":ti,ab,kw OR ((Fort:ti,ab,kw OR Ft:ti,ab,kw) NEXT Collins:ti,ab,kw) OR Lancaster:ti,ab,kw OR Palmdale:ti,ab,kw OR Murfreesboro:ti,ab,kw OR Salinas:ti,ab,kw OR Corona:ti,ab,kw OR Killeen:ti,ab,kw OR Hayward:ti,ab,kw OR Paterson:ti,ab,kw OR Macon:ti,ab,kw OR Lakewood:ti,ab,kw OR Alexandria:ti,ab,kw OR Roseville:ti,ab,kw OR Surprise:ti,ab,kw OR Springfield:ti,ab,kw OR Charleston:ti,ab,kw OR "Kansas City":ti,ab,kw OR Sunnyvale:ti,ab,kw OR Bellevue:ti,ab,kw OR Hollywood:ti,ab,kw OR Denton:ti,ab,kw OR Escondido:ti,ab,kw OR Joliet:ti,ab,kw OR Naperville:ti,ab,kw OR Bridgeport:ti,ab,kw OR Savannah:ti,ab,kw OR Mesquite:ti,ab,kw OR Pasadena:ti,ab,kw OR Rockford:ti,ab,kw OR Pomona:ti,ab,kw OR Jackson:ti,ab,kw OR Olathe:ti,ab,kw OR Gainesville:ti,ab,kw OR McAllen:ti,ab,kw OR Syracuse:ti,ab,kw OR Waco:ti,ab,kw OR Visalia:ti,ab,kw OR Thornton:ti,ab,kw OR Torrance:ti,ab,kw OR Fullerton:ti,ab,kw OR Columbia:ti,ab,kw OR Lakewood:ti,ab,kw OR "New Haven":ti,ab,kw OR Hampton:ti,ab,kw OR Miramar:ti,ab,kw OR Victorville:ti,ab,kw OR Warren:ti,ab,kw OR "West Valley City":ti,ab,kw OR "Cedar Rapids":ti,ab,kw OR Stamford:ti,ab,kw OR Orange:ti,ab,kw OR Dayton:ti,ab,kw OR Midland:ti,ab,kw OR Kent:ti,ab,kw OR Elizabeth:ti,ab,kw OR Pasadena:ti,ab,kw OR Carrollton:ti,ab,kw OR "Coral Springs":ti,ab,kw OR "Sterling Heights":ti,ab,kw OR Fargo:ti,ab,kw OR Lewisville:ti,ab,kw OR Meridian:ti,ab,kw OR Norman:ti,ab,kw OR "Palm Bay":ti,ab,kw OR Athens:ti,ab,kw OR Columbia:ti,ab,kw OR Abilene:ti,ab,kw OR Pearland:ti,ab,kw OR "Santa Clara":ti,ab,kw OR "Round Rock":ti,ab,kw OR Topeka:ti,ab,kw OR Allentown:ti,ab,kw OR Clovis:ti,ab,kw OR "Simi Valley":ti,ab,kw OR "College Station":ti,ab,kw OR "Thousand Oaks":ti,ab,kw OR Vallejo:ti,ab,kw OR Concord:ti,ab,kw OR Rochester:ti,ab,kw OR Arvada:ti,ab,kw OR Lafayette:ti,ab,kw OR Independence:ti,ab,kw OR "West Palm Beach":ti,ab,kw OR Hartford:ti,ab,kw OR Wilmington:ti,ab,kw OR Lakeland:ti,ab,kw OR Billings:ti,ab,kw OR "Ann Arbor":ti,ab,kw OR Fairfield:ti,ab,kw OR Berkeley:ti,ab,kw OR Richardson:ti,ab,kw OR "North Charleston":ti,ab,kw OR Cambridge:ti,ab,kw OR "Broken Arrow":ti,ab,kw OR Clearwater:ti,ab,kw OR "West Jordan":ti,ab,kw OR Evansville:ti,ab,kw OR "League City":ti,ab,kw OR Antioch:ti,ab,kw OR Manchester:ti,ab,kw OR "High Point":ti,ab,kw OR Waterbury:ti,ab,kw OR Westminster:ti,ab,kw OR Richmond:ti,ab,kw OR Carlsbad:ti,ab,kw OR "Las Cruces":ti,ab,kw OR Murrieta:ti,ab,kw OR Lowell:ti,ab,kw OR Provo:ti,ab,kw OR Springfield:ti,ab,kw OR Elgin:ti,ab,kw OR Odessa:ti,ab,kw OR Lansing:ti,ab,kw OR "Pompano Beach":ti,ab,kw OR Beaumont:ti,ab,kw OR Temecula:ti,ab,kw OR Gresham:ti,ab,kw OR Allen:ti,ab,kw OR Pueblo:ti,ab,kw OR Everett:ti,ab,kw OR "South Fulton":ti,ab,kw OR Peoria:ti,ab,kw OR Nampa:ti,ab,kw OR Tuscaloosa:ti,ab,kw OR "Miami Gardens":ti,ab,kw OR "Santa Maria":ti,ab,kw OR Downey:ti,ab,kw OR Concord:ti,ab,kw OR Ventura:ti,ab,kw OR "Costa Mesa":ti,ab,kw OR "Sugar Land":ti,ab,kw OR Menifee:ti,ab,kw OR Tyler:ti,ab,kw OR Sparks:ti,ab,kw OR Greeley:ti,ab,kw OR "Rio Rancho":ti,ab,kw OR "Sandy Springs":ti,ab,kw OR Dearborn:ti,ab,kw OR "Jurupa Valley":ti,ab,kw OR Edison:ti,ab,kw OR "Spokane Valley":ti,ab,kw OR Hillsboro:ti,ab,kw OR Davie:ti,ab,kw OR "Green Bay":ti,ab,kw OR Centennial:ti,ab,kw OR Buckeye:ti,ab,kw OR Boulder:ti,ab,kw OR Goodyear:ti,ab,kw OR "El Monte":ti,ab,kw OR "West Covina":ti,ab,kw OR Brockton:ti,ab,kw OR "New Braunfels":ti,ab,kw OR "El Cajon":ti,ab,kw OR Edinburg:ti,ab,kw OR Renton:ti,ab,kw OR Burbank:ti,ab,kw OR Inglewood:ti,ab,kw OR Rialto:ti,ab,kw OR "Lee's Summit":ti,ab,kw OR Bend:ti,ab,kw OR Woodbridge:ti,ab,kw OR "South Bend":ti,ab,kw OR "Wichita Falls":ti,ab,kw OR ((Saint:ti,ab,kw OR St:ti,ab,kw) NEXT George:ti,ab,kw) OR Fishers:ti,ab,kw OR Carmel:ti,ab,kw OR Vacaville:ti,ab,kw OR Quincy:ti,ab,kw OR Conroe:ti,ab,kw OR Chico:ti,ab,kw OR "San Mateo":ti,ab,kw OR Lynn:ti,ab,kw OR Albany:ti,ab,kw OR Hesperia:ti,ab,kw OR "New Bedford":ti,ab,kw OR Davenport:ti,ab,kw OR "Daly City":ti,ab,kw) |
| #79 | {OR #75-#78} |
| #80 | #69 OR (#74 AND #79) |
| #81 | #8 AND #68 AND #80 in Trials |

**Scopus (Elsevier)
Searched February 9, 2024.
208 records retrieved.**

| ( TITLE-ABS-KEY ( ( diabet* W/2 ( adult-onset OR insulin-independent* OR ketosis-resistant OR latent* OR matur*-onset OR non-insulin-dependent OR slow-onset OR stable OR type-2 OR type-ii ) ) OR ( dm-2* OR dm2* OR mody* OR niddm* OR t2d* OR t2dm* ) OR ( ( metabolic* OR cardiometabolic OR cardio-metabolic* OR dysmetabolic* OR dys-metabolic* OR insulin-resistan* OR reaven* ) W/3 syndrom* ) OR ( a1c OR hb-a1c OR haemoglobin-a1c OR haemoglobin-a-1c OR haemoglobin-aic OR hba-1c OR hba1c OR hemoglobin-a1c OR hemoglobin-a-1c OR hemoglobin-aic ) OR ( glucosaemi* OR glucosemi* OR glycaemi* OR glycemi* OR hyperglycaemi* OR hyperglycemia* OR hyper-glycemia* OR hyperglycaemi* OR hyper-glycaemi* OR hyperglucemi* ) ) )  AND  ( TITLE-ABS-KEY ( ( intraop* OR intra-op* OR intrasurg* OR intra-surg* ) OR ( ( oper* OR surg* ) W/2 ( durat* OR length* OR time* ) ) OR ( postop* OR post-op* OR postsurg* OR post-surg* OR post-proced* ) OR ( ( operat* OR surg* OR proced* ) W/7 sequelae* ) OR ( periop* OR peri-op* OR perisurg* OR peri-surg* ) OR ( anesthe* OR anaesthe* OR cryoanaesthe* OR cryo-anaesthe* OR cryoanesthe* OR cryo-anesthe* OR electro-anaesthe* OR electroanasthaesia OR electroanesthe* OR electro-anesthe* OR neuro-anaesthe* OR neuroanasthaesia OR neuroanesthe* OR neuro-anesthe* OR neuroleptanaesthe* OR neuro-leptanaesthe* OR neuroleptanesthe* OR neuro-leptanesthe* ) OR ( block* W/3 ( autonomic OR cauda OR caudal OR cervical OR conduction OR dural OR epidural OR extradural OR ganglion OR intercostal OR lumbar OR nerve OR neurogenic OR neuromuscular OR paracervical OR peridural OR "ql" OR "quadratus lumborum" OR retrobulbar OR retroocular OR spinal OR stellate-ganglion OR subarachnoid OR tap OR "transverse abdominal plane" ) ) OR ( postanesthes* OR post-anesthes* OR postanaesthes* OR post-anesthaes* OR ( recover* W/1 ( room* OR ward* OR period* ) ) OR pacu ) OR ( ( surg* OR operat* ) W/2 ( advers* OR injur* OR complicat* OR blood-loss OR hemorrhag* OR haemorrhag* ) ) OR ( ( anesthesi* OR anesthaesi* OR malignan* ) W/4 ( hypertherm* OR hyperpyrex* ) ) OR ( ( afferent* OR efferent* ) W/1 ( loop* OR limb* ) W/3 ( syndrome* OR disease* OR obstruct* ) ) OR ( ( anastomosis* OR anastomotic* ) W/3 ( dehiscen* OR leak* OR rupture* ) ) OR ( ( ( breast* W/2 cancer ) OR mastectom* ) W/3 ( lymphedem* OR lymphoedem* ) ) OR ( cornea* W/1 endothel* W/2 ( loss* OR damag* OR injur* ) ) OR ( "coronary-subclavian steal" ) OR ( ( delay* W/2 ( awake* OR conscious* OR ( emergenc* W/2 anesth*es* ) ) ) OR ( residual W/2 ( block* OR curarizat* ) ) ) OR ( ( emerg* OR postanesthes* OR post-anesthes* OR postanaesthes* OR post-anesthaes* ) W/1 ( deliri* OR agitat* OR excit* ) ) OR ( "failed back surg*" OR ( ( failed-back OR post-laminectom* OR postlaminectom* ) W/2 syndrome* ) OR fbss ) OR ( ( graft* OR allograft* OR transplant* OR flap* ) W/2 ( occlusion* OR occlude* OR restenosi* OR disease* OR disorder* OR dysfunct* OR fail* OR infect* OR necros* OR reject* OR vasculopath* ) ) OR ( ( cicatr* OR incision* OR scar* ) W/3 hernia* ) OR ( pseudomeli* OR pseudo-meli* OR ( phantom W/3 ( limb* OR pain* OR sensation* ) ) ) OR ( cholecystectom* OR post-cholecystectom* OR postcholecystectom* OR choledochoduodenostom* OR post-choledochoduodenostom* OR postcholedochoduodenostom* OR sump ) OR ( postgastrectom* OR post-gastrectom* OR stomach-resect* OR dumping* ) OR ( endoleak* OR endo-leak* OR perigraft-leak* ) OR ( ponv ) OR ( postcommissurotom* OR postpericardiotom* OR post-commissurotom* OR post-pericardiotom* ) OR ( ( prosthesis* OR prosthetic* ) W/3 ( fail* OR complicat* OR durab* OR loos* OR migrat* OR infect* OR surviv* ) ) OR ( "capsul* contract*" ) OR ( reperfus* W/3 ( injur* OR damag* ) ) OR ( ( "post-cardiac arrest*" OR postresuscitation OR post-resuscitat* OR post-heart-arrest* ) W/3 syndrome* ) OR ( "primary graft dysfunct*" ) OR ( surg* W/3 ( shock OR collaps* ) ) OR ( short* W/1 ( bowel* OR gut* OR intestin* ) W/1 syndrome* ) OR ( slit-ventricle* ) OR ( ( wound* OR suture* OR surgical-wound* ) W/3 ( dehiscence* OR disrupt* OR rupture* OR separat* OR seperat* ) ) OR ( vasopleg* OR vaso-pleg* ) OR ( bone* W/3 ( cement* OR glue* OR paste* ) ) OR ( ( breast* OR mammar* ) W/3 ( implant* OR prosthes* OR endoprosthes ) ) OR ( diffus* W/2 lamellar* W/2 keratit* ) OR ( gastric*-band* W/3 ( erode* OR erosion* OR fail* ) ) OR ( hypocalcemi* OR hypocalcaemi* OR hungry-bone* ) OR ( ( low-anterior-resection OR lar ) W/3 syndrome* ) OR ( paravalvular W/1 ( leak* OR regurgitat* ) ) OR ( malignan*-glaucoma OR aqueous-misdirection OR ciliar*-block-glaucoma ) OR ( pseudo* W/3 glaucoma ) OR ( ( pneumonectom* OR post-pneumonectom* OR postpneumonectom* ) W/3 syndrome* ) OR ( ( endoprosthe* OR endo-prosthe* OR prosthe* OR periprosthe* OR peri-prosthe* OR screw* ) W/3 ( dysfunction* OR disfunction* OR deteriorat* OR dislocat* OR endocarditis* OR erod* OR erosion* OR fail* OR fracture* OR loosen* OR thrombosis ) ) OR ( ( retain* W/3 ( appliance* OR body OR bodies OR clip* OR cotton* OR drain* OR gauze* OR linen* OR item* OR instrument* OR material* OR needle* OR object* OR pad OR pads OR sponge* OR swab* OR textile* OR tool* OR towel* ) ) OR textiloma* OR gossypiboma* ) OR ( ( surg* OR operat* ) W/3 infect* ) OR ( scar* W/3 endometriosis* ) ) )  AND  ( TITLE-ABS-KEY ( ainu* OR asia* OR bai* OR bangladesh* OR bhote* OR bhutan* OR bornean* OR borneo* OR brunei* OR burma* OR burmese* OR cambodia* OR ceylon* OR china* OR chinese* OR darussalam* OR dayak* OR dyak* OR filipin* OR hmong* OR "hong kong*" OR hongkong* OR hu OR huren OR huzu OR hus OR india* OR indo-chin* OR indonesia* OR iu-mien* OR japan* OR kalmyk* OR kampuchea* OR kampudja* OR karen* OR kashmir* OR khmer* OR korea* OR lao* OR macanes* OR macanese* OR macao* OR macau* OR malaysia* OR maldiv* OR manchu* OR maylay* OR melanesia* OR mien* OR mongol* OR mongolia* OR myanma* OR nepal* OR pakistan* OR paracel* OR philippin* OR ryukyuan* OR singapor* OR sinhal* OR south*-asia* OR spratly* OR srilanka* OR sri-lanka* OR taiwan* OR tamil* OR thai* OR tibet* OR timor* OR turkic* OR viet* OR zhuang* ) ) AND ( TITLE-ABS-KEY ( ( america* OR "united states" ) OR ( alabama* OR alaska* OR arizona* OR arkansa* OR california* OR carolina* OR colorad* OR connecticut* OR dakota* OR delaware* OR "district of columbia" OR florida* OR floridian* OR georgia* OR hawaii* OR idaho* OR illinois* OR indiana* OR iowa* OR kansas OR kansan OR kentuck* OR louisiana* OR maine* OR maryland* OR massachusetts* OR michigan* OR minnesota* OR mississippi* OR missouri* OR montana* OR nebraska* OR nevada* OR "new england*" OR "new hampshir*" OR "new jersey*" OR "new mexic*" OR "new york*" OR "north carolin*" OR ohio* OR oklahom* OR oregon* OR pennsylvania* OR "puerto ric*" OR "rhode island*" OR "south carolin*" OR tennesse* OR texas* OR texan* OR utah* OR vermont* OR virginia* OR washington* OR "west virginia*" OR wisconsin* OR wyoming* ) OR ( "new york" OR "los angeles" OR chicago OR houston OR phoenix OR philadelphia OR "san antonio" OR "san diego" OR dallas OR austin OR jacksonville OR "san jose" OR ( ( fort OR ft ) W/1 worth ) OR columbus OR charlotte OR indianapolis OR "san francisco" OR seattle OR denver OR "oklahoma city" OR nashville OR "el paso" OR washington OR "las vegas" OR boston OR portland OR louisville OR memphis OR detroit OR baltimore OR milwaukee OR albuquerque OR tucson OR fresno OR sacramento OR mesa OR "kansas city" OR atlanta OR "colorado springs" OR omaha OR raleigh OR "virginia beach" OR "long beach" OR miami OR oakland OR minneapolis OR tulsa OR bakersfield OR tampa OR wichita OR arlington OR aurora OR "new orleans" OR cleveland OR anaheim OR honolulu OR henderson OR stockton OR riverside OR lexington OR "corpus christi" OR orlando OR irvine OR cincinnati OR "santa ana" OR newark OR ( ( saint OR st ) W/1 paul ) OR pittsburgh OR greensboro OR lincoln OR durham OR plano OR anchorage OR "jersey city" OR ( ( saint OR st ) W/1 louis ) OR chandler OR "north las vegas" OR "chula vista" OR buffalo OR gilbert OR reno OR madison OR ( ( fort OR ft ) W/1 wayne ) OR toledo OR lubbock OR ( ( saint OR st ) W/1 petersburg ) OR laredo OR irving OR chesapeake OR glendale OR winston-salem OR scottsdale OR garland OR boise OR norfolk OR ( port W/1 ( saint OR st ) W/1 lucie ) OR spokane OR richmond OR fremont OR huntsville OR tacoma OR "baton rouge" OR "santa clarita" OR "san bernardino" OR hialeah OR frisco OR modesto OR "cape coral" OR fontana OR "moreno valley" OR "des moines" OR rochester OR fayetteville OR yonkers OR mckinney OR worcester OR "salt lake city" OR "little rock" OR columbus OR augusta OR "sioux falls" OR "grand prairie" OR tallahassee OR amarillo OR oxnard OR peoria OR "overland park" OR montgomery OR birmingham OR "grand rapids" OR knoxville OR vancouver OR "huntington beach" OR providence OR brownsville OR glendale OR akron OR tempe OR "newport news" OR chattanooga OR mobile OR ( ( fort OR ft ) W/1 lauderdale ) OR cary OR shreveport OR ontario OR eugene OR aurora OR "elk grove" OR salem OR "santa rosa" OR clarksville OR "rancho cucamonga" OR oceanside OR springfield OR "pembroke pines" OR "garden grove" OR ( ( fort OR ft ) W/1 collins ) OR lancaster OR palmdale OR murfreesboro OR salinas OR corona OR killeen OR hayward OR paterson OR macon OR lakewood OR alexandria OR roseville OR surprise OR springfield OR charleston OR "kansas city" OR sunnyvale OR bellevue OR hollywood OR denton OR escondido OR joliet OR naperville OR bridgeport OR savannah OR mesquite OR pasadena OR rockford OR pomona OR jackson OR olathe OR gainesville OR mcallen OR syracuse OR waco OR visalia OR thornton OR torrance OR fullerton OR columbia OR lakewood OR "new haven" OR hampton OR miramar OR victorville OR warren OR "west valley city" OR "cedar rapids" OR stamford OR orange OR dayton OR midland OR kent OR elizabeth OR pasadena OR carrollton OR "coral springs" OR "sterling heights" OR fargo OR lewisville OR meridian OR norman OR "palm bay" OR athens OR columbia OR abilene OR pearland OR "santa clara" OR "round rock" OR topeka OR allentown OR clovis OR "simi valley" OR "college station" OR "thousand oaks" OR vallejo OR concord OR rochester OR arvada OR lafayette OR independence OR "west palm beach" OR hartford OR wilmington OR lakeland OR billings OR "ann arbor" OR fairfield OR berkeley OR richardson OR "north charleston" OR cambridge OR "broken arrow" OR clearwater OR "west jordan" OR evansville OR "league city" OR antioch OR manchester OR "high point" OR waterbury OR westminster OR richmond OR carlsbad OR "las cruces" OR murrieta OR lowell OR provo OR springfield OR elgin OR odessa OR lansing OR "pompano beach" OR beaumont OR temecula OR gresham OR allen OR pueblo OR everett OR "south fulton" OR peoria OR nampa OR tuscaloosa OR "miami gardens" OR "santa maria" OR downey OR concord OR ventura OR "costa mesa" OR "sugar land" OR menifee OR tyler OR sparks OR greeley OR "rio rancho" OR "sandy springs" OR dearborn OR "jurupa valley" OR edison OR "spokane valley" OR hillsboro OR davie OR "green bay" OR centennial OR buckeye OR boulder OR goodyear OR "el monte" OR "west covina" OR brockton OR "new braunfels" OR "el cajon" OR edinburg OR renton OR burbank OR inglewood OR rialto OR "lee&apos;s summit" OR bend OR woodbridge OR "south bend" OR "wichita falls" OR ( ( saint OR st ) W/1 george ) OR fishers OR carmel OR vacaville OR quincy OR conroe OR chico OR "san mateo" OR lynn OR albany OR hesperia OR "new bedford" OR davenport OR "daly city" ) ) ) |
| --- |

**Web of Science (Clarivate)
Searched February 9, 2024.
224 records retrieved.**
Databases: Web of Science Core Collection: Science Citation Index Expanded (1945-present), Social Sciences Citation Index (1956-present), Arts & Humanities Citation Index (1975-present), Conference Proceedings Citation Index- Science (1990-present), Conference Proceedings Citation Index- Social Science & Humanities (1990-present), Emerging Sources Citation Index (2015-present).
KCI-Korean Journal Database, (1980-present); SciELO Citation Index, (2002-present); Preprint Citation Index, (1991-present); ProQuest Dissertations & Theses Citation Index, (1637-present).
Timespan=1945-2021
Language = Auto

| ( TS= ( ( diabet* NEAR/2 ( adult-onset OR insulin-independent* OR ketosis-resistant OR latent* OR matur*-onset OR non-insulin-dependent OR slow-onset OR stable OR type-2 OR type-ii ) ) OR ( dm-2* OR dm2* OR mody* OR niddm* OR t2d* OR t2dm* ) OR ( ( metabolic* OR cardiometabolic OR cardio-metabolic* OR dysmetabolic* OR dys-metabolic* OR insulin-resistan* OR reaven* ) NEAR/3 syndrom* ) OR ( a1c OR hb-a1c OR haemoglobin-a1c OR haemoglobin-a-1c OR haemoglobin-aic OR hba-1c OR hba1c OR hemoglobin-a1c OR hemoglobin-a-1c OR hemoglobin-aic ) OR ( glucosaemi* OR glucosemi* OR glycaemi* OR glycemi* OR hyperglycaemi* OR hyperglycemia* OR hyper-glycemia* OR hyperglycaemi* OR hyper-glycaemi* OR hyperglucemi* ) ) )  AND  ( TS= ( ( intraop* OR intra-op* OR intrasurg* OR intra-surg* ) OR ( ( oper* OR surg* ) NEAR/2 ( durat* OR length* OR time* ) ) OR ( postop* OR post-op* OR postsurg* OR post-surg* OR post-proced* ) OR ( ( operat* OR surg* OR proced* ) NEAR/7 sequelae* ) OR ( periop* OR peri-op* OR perisurg* OR peri-surg* ) OR ( anesthe* OR anaesthe* OR cryoanaesthe* OR cryo-anaesthe* OR cryoanesthe* OR cryo-anesthe* OR electro-anaesthe* OR electroanasthaesia OR electroanesthe* OR electro-anesthe* OR neuro-anaesthe* OR neuroanasthaesia OR neuroanesthe* OR neuro-anesthe* OR neuroleptanaesthe* OR neuro-leptanaesthe* OR neuroleptanesthe* OR neuro-leptanesthe* ) OR ( block* NEAR/3 ( autonomic OR cauda OR caudal OR cervical OR conduction OR dural OR epidural OR extradural OR ganglion OR intercostal OR lumbar OR nerve OR neurogenic OR neuromuscular OR paracervical OR peridural OR "ql" OR "quadratus lumborum" OR retrobulbar OR retroocular OR spinal OR stellate-ganglion OR subarachnoid OR tap OR "transverse abdominal plane" ) ) OR ( postanesthes* OR post-anesthes* OR postanaesthes* OR post-anesthaes* OR ( recover* NEAR/1 ( room* OR ward* OR period* ) ) OR pacu ) OR ( ( surg* OR operat* ) NEAR/2 ( advers* OR injur* OR complicat* OR blood-loss OR hemorrhag* OR haemorrhag* ) ) OR ( ( anesthesi* OR anesthaesi* OR malignan* ) NEAR/4 ( hypertherm* OR hyperpyrex* ) ) OR ( ( afferent* OR efferent* ) NEAR/1 ( loop* OR limb* ) NEAR/3 ( syndrome* OR disease* OR obstruct* ) ) OR ( ( anastomosis* OR anastomotic* ) NEAR/3 ( dehiscen* OR leak* OR rupture* ) ) OR ( ( ( breast* NEAR/2 cancer ) OR mastectom* ) NEAR/3 ( lymphedem* OR lymphoedem* ) ) OR ( cornea* NEAR/1 endothel* NEAR/2 ( loss* OR damag* OR injur* ) ) OR ( "coronary-subclavian steal" ) OR ( ( delay* NEAR/2 ( awake* OR conscious* OR ( emergenc* NEAR/2 anesth*es* ) ) ) OR ( residual NEAR/2 ( block* OR curarizat* ) ) ) OR ( ( emerg* OR postanesthes* OR post-anesthes* OR postanaesthes* OR post-anesthaes* ) NEAR/1 ( deliri* OR agitat* OR excit* ) ) OR ( "failed back surg*" OR ( ( failed-back OR post-laminectom* OR postlaminectom* ) NEAR/2 syndrome* ) OR fbss ) OR ( ( graft* OR allograft* OR transplant* OR flap* ) NEAR/2 ( occlusion* OR occlude* OR restenosi* OR disease* OR disorder* OR dysfunct* OR fail* OR infect* OR necros* OR reject* OR vasculopath* ) ) OR ( ( cicatr* OR incision* OR scar* ) NEAR/3 hernia* ) OR ( pseudomeli* OR pseudo-meli* OR ( phantom NEAR/3 ( limb* OR pain* OR sensation* ) ) ) OR ( cholecystectom* OR post-cholecystectom* OR postcholecystectom* OR choledochoduodenostom* OR post-choledochoduodenostom* OR postcholedochoduodenostom* OR sump ) OR ( postgastrectom* OR post-gastrectom* OR stomach-resect* OR dumping* ) OR ( endoleak* OR endo-leak* OR perigraft-leak* ) OR ( ponv ) OR ( postcommissurotom* OR postpericardiotom* OR post-commissurotom* OR post-pericardiotom* ) OR ( ( prosthesis* OR prosthetic* ) NEAR/3 ( fail* OR complicat* OR durab* OR loos* OR migrat* OR infect* OR surviv* ) ) OR ( "capsul* contract*" ) OR ( reperfus* NEAR/3 ( injur* OR damag* ) ) OR ( ( "post-cardiac arrest*" OR postresuscitation OR post-resuscitat* OR post-heart-arrest* ) NEAR/3 syndrome* ) OR ( "primary graft dysfunct*" ) OR ( surg* NEAR/3 ( shock OR collaps* ) ) OR ( short* NEAR/1 ( bowel* OR gut* OR intestin* ) NEAR/1 syndrome* ) OR ( slit-ventricle* ) OR ( ( wound* OR suture* OR surgical-wound* ) NEAR/3 ( dehiscence* OR disrupt* OR rupture* OR separat* OR seperat* ) ) OR ( vasopleg* OR vaso-pleg* ) OR ( bone* NEAR/3 ( cement* OR glue* OR paste* ) ) OR ( ( breast* OR mammar* ) NEAR/3 ( implant* OR prosthes* OR endoprosthes ) ) OR ( diffus* NEAR/2 lamellar* NEAR/2 keratit* ) OR ( gastric*-band* NEAR/3 ( erode* OR erosion* OR fail* ) ) OR ( hypocalcemi* OR hypocalcaemi* OR hungry-bone* ) OR ( ( low-anterior-resection OR lar ) NEAR/3 syndrome* ) OR ( paravalvular NEAR/1 ( leak* OR regurgitat* ) ) OR ( malignan*-glaucoma OR aqueous-misdirection OR ciliar*-block-glaucoma ) OR ( pseudo* NEAR/3 glaucoma ) OR ( ( pneumonectom* OR post-pneumonectom* OR postpneumonectom* ) NEAR/3 syndrome* ) OR ( ( endoprosthe* OR endo-prosthe* OR prosthe* OR periprosthe* OR peri-prosthe* OR screw* ) NEAR/3 ( dysfunction* OR disfunction* OR deteriorat* OR dislocat* OR endocarditis* OR erod* OR erosion* OR fail* OR fracture* OR loosen* OR thrombosis ) ) OR ( ( retain* NEAR/3 ( appliance* OR body OR bodies OR clip* OR cotton* OR drain* OR gauze* OR linen* OR item* OR instrument* OR material* OR needle* OR object* OR pad OR pads OR sponge* OR swab* OR textile* OR tool* OR towel* ) ) OR textiloma* OR gossypiboma* ) OR ( ( surg* OR operat* ) NEAR/3 infect* ) OR ( scar* NEAR/3 endometriosis* ) ) )  AND  ( TS= ( ainu* OR asia* OR bai* OR bangladesh* OR bhote* OR bhutan* OR bornean* OR borneo* OR brunei* OR burma* OR burmese* OR cambodia* OR ceylon* OR china* OR chinese* OR darussalam* OR dayak* OR dyak* OR filipin* OR hmong* OR "hong kong*" OR hongkong* OR hu OR huren OR huzu OR hus OR india* OR indo-chin* OR indonesia* OR iu-mien* OR japan* OR kalmyk* OR kampuchea* OR kampudja* OR karen* OR kashmir* OR khmer* OR korea* OR lao* OR macanes* OR macanese* OR macao* OR macau* OR malaysia* OR maldiv* OR manchu* OR maylay* OR melanesia* OR mien* OR mongol* OR mongolia* OR myanma* OR nepal* OR pakistan* OR paracel* OR philippin* OR ryukyuan* OR singapor* OR sinhal* OR south*-asia* OR spratly* OR srilanka* OR sri-lanka* OR taiwan* OR tamil* OR thai* OR tibet* OR timor* OR turkic* OR viet* OR zhuang* ) ) AND ( TS= ( ( america* OR "united states" ) OR ( alabama* OR alaska* OR arizona* OR arkansa* OR california* OR carolina* OR colorad* OR connecticut* OR dakota* OR delaware* OR "district of columbia" OR florida* OR floridian* OR georgia* OR hawaii* OR idaho* OR illinois* OR indiana* OR iowa* OR kansas OR kansan OR kentuck* OR louisiana* OR maine* OR maryland* OR massachusetts* OR michigan* OR minnesota* OR mississippi* OR missouri* OR montana* OR nebraska* OR nevada* OR "new england*" OR "new hampshir*" OR "new jersey*" OR "new mexic*" OR "new york*" OR "north carolin*" OR ohio* OR oklahom* OR oregon* OR pennsylvania* OR "puerto ric*" OR "rhode island*" OR "south carolin*" OR tennesse* OR texas* OR texan* OR utah* OR vermont* OR virginia* OR washington* OR "west virginia*" OR wisconsin* OR wyoming* ) OR ( "new york" OR "los angeles" OR chicago OR houston OR phoenix OR philadelphia OR "san antonio" OR "san diego" OR dallas OR austin OR jacksonville OR "san jose" OR ( ( fort OR ft ) NEAR/1 worth ) OR columbus OR charlotte OR indianapolis OR "san francisco" OR seattle OR denver OR "oklahoma city" OR nashville OR "el paso" OR washington OR "las vegas" OR boston OR portland OR louisville OR memphis OR detroit OR baltimore OR milwaukee OR albuquerque OR tucson OR fresno OR sacramento OR mesa OR "kansas city" OR atlanta OR "colorado springs" OR omaha OR raleigh OR "virginia beach" OR "long beach" OR miami OR oakland OR minneapolis OR tulsa OR bakersfield OR tampa OR wichita OR arlington OR aurora OR "new orleans" OR cleveland OR anaheim OR honolulu OR henderson OR stockton OR riverside OR lexington OR "corpus christi" OR orlando OR irvine OR cincinnati OR "santa ana" OR newark OR ( ( saint OR st ) NEAR/1 paul ) OR pittsburgh OR greensboro OR lincoln OR durham OR plano OR anchorage OR "jersey city" OR ( ( saint OR st ) NEAR/1 louis ) OR chandler OR "north las vegas" OR "chula vista" OR buffalo OR gilbert OR reno OR madison OR ( ( fort OR ft ) NEAR/1 wayne ) OR toledo OR lubbock OR ( ( saint OR st ) NEAR/1 petersburg ) OR laredo OR irving OR chesapeake OR glendale OR winston-salem OR scottsdale OR garland OR boise OR norfolk OR ( port NEAR/1 ( saint OR st ) NEAR/1 lucie ) OR spokane OR richmond OR fremont OR huntsville OR tacoma OR "baton rouge" OR "santa clarita" OR "san bernardino" OR hialeah OR frisco OR modesto OR "cape coral" OR fontana OR "moreno valley" OR "des moines" OR rochester OR fayetteville OR yonkers OR mckinney OR worcester OR "salt lake city" OR "little rock" OR columbus OR augusta OR "sioux falls" OR "grand prairie" OR tallahassee OR amarillo OR oxnard OR peoria OR "overland park" OR montgomery OR birmingham OR "grand rapids" OR knoxville OR vancouver OR "huntington beach" OR providence OR brownsville OR glendale OR akron OR tempe OR "newport news" OR chattanooga OR mobile OR ( ( fort OR ft ) NEAR/1 lauderdale ) OR cary OR shreveport OR ontario OR eugene OR aurora OR "elk grove" OR salem OR "santa rosa" OR clarksville OR "rancho cucamonga" OR oceanside OR springfield OR "pembroke pines" OR "garden grove" OR ( ( fort OR ft ) NEAR/1 collins ) OR lancaster OR palmdale OR murfreesboro OR salinas OR corona OR killeen OR hayward OR paterson OR macon OR lakewood OR alexandria OR roseville OR surprise OR springfield OR charleston OR "kansas city" OR sunnyvale OR bellevue OR hollywood OR denton OR escondido OR joliet OR naperville OR bridgeport OR savannah OR mesquite OR pasadena OR rockford OR pomona OR jackson OR olathe OR gainesville OR mcallen OR syracuse OR waco OR visalia OR thornton OR torrance OR fullerton OR columbia OR lakewood OR "new haven" OR hampton OR miramar OR victorville OR warren OR "west valley city" OR "cedar rapids" OR stamford OR orange OR dayton OR midland OR kent OR elizabeth OR pasadena OR carrollton OR "coral springs" OR "sterling heights" OR fargo OR lewisville OR meridian OR norman OR "palm bay" OR athens OR columbia OR abilene OR pearland OR "santa clara" OR "round rock" OR topeka OR allentown OR clovis OR "simi valley" OR "college station" OR "thousand oaks" OR vallejo OR concord OR rochester OR arvada OR lafayette OR independence OR "west palm beach" OR hartford OR wilmington OR lakeland OR billings OR "ann arbor" OR fairfield OR berkeley OR richardson OR "north charleston" OR cambridge OR "broken arrow" OR clearwater OR "west jordan" OR evansville OR "league city" OR antioch OR manchester OR "high point" OR waterbury OR westminster OR richmond OR carlsbad OR "las cruces" OR murrieta OR lowell OR provo OR springfield OR elgin OR odessa OR lansing OR "pompano beach" OR beaumont OR temecula OR gresham OR allen OR pueblo OR everett OR "south fulton" OR peoria OR nampa OR tuscaloosa OR "miami gardens" OR "santa maria" OR downey OR concord OR ventura OR "costa mesa" OR "sugar land" OR menifee OR tyler OR sparks OR greeley OR "rio rancho" OR "sandy springs" OR dearborn OR "jurupa valley" OR edison OR "spokane valley" OR hillsboro OR davie OR "green bay" OR centennial OR buckeye OR boulder OR goodyear OR "el monte" OR "west covina" OR brockton OR "new braunfels" OR "el cajon" OR edinburg OR renton OR burbank OR inglewood OR rialto OR "lee&apos;s summit" OR bend OR woodbridge OR "south bend" OR "wichita falls" OR ( ( saint OR st ) NEAR/1 george ) OR fishers OR carmel OR vacaville OR quincy OR conroe OR chico OR "san mateo" OR lynn OR albany OR hesperia OR "new bedford" OR davenport OR "daly city" ) ) ) |
| --- |

John M. Reynolds, MLIS
University of Miami Miller School of Medicine
Calder Memorial Library
https://orcid.org/0000-0001-6744-3517
