# Supplementary material for: Perioperative Risks and Outcomes in Asian American Patients with Type 2 Diabetes Mellitus and/or Metabolic Syndrome: a Systematic Scoping Review
Source: J Racial Ethn Health Disparities. 2025 Mar 5;13(2):1417–33. doi: 10.1007/s40615-025-02344-6 (PMC12966264; doi:10.1007/s40615-025-02344-6)
Supplement: Supplementary file 2 — Supplementary file2 (DOCX 25 kb) [file 40615_2025_2344_MOESM2_ESM.docx]

Appendix 1: Studies excluded after reading full text

Abugo U, Jones LS, Kwagyan J. The influence of diabetes on glaucoma surgical outcomes in the minority population. *Invest Ophthalmol Vis Sci.* 2015;56(7):2661.

Exclusion reason: Abstract only - insufficient data

Ali H, Patel P, Pamarthy R, et al. Racial disparities in endoscopic sleeve gastroplasty in the United States. *Gastrointest Endosc.* 2023;97(6):AB26.

Exclusion reason: Abstract only - insufficient data

Ananth J. Atypical antipsychotic drugs, diabetes and ethnicity. *Expert Opin Drug Saf.* 2005;4(6):1111-1124.

Exclusion reason: Abstract only - insufficient data

Dixon J, Eaton L, Vincent V, Michaelson R. LAP-BAND® for BMI 30-40: 5-year health outcomes from the multicenter pivotal study. *Obes Surg.* 2015;25(8):1307.

Exclusion reason: Abstract only - insufficient data

Gruenbaum S, Guay C, Konkayev A, et al. Perioperative glycemia management in patients undergoing craniotomy for brain tumor resection: An international survey of neuroanesthesiologists' perceptions and practices. *J Neurosurg Anesthesiol.* 2019;31(4):466.

Exclusion reason: Abstract only - insufficient data

Hampton LA, Mocanu V, Verhoeff K, Birch DW, Karmali S, Switzer NJ. Characterizing the role of Asian racial status in post-operative complications and mortality in patients undergoing elective bariatric surgery: An MBSAQIP analysis of 594 837 patients. *Surg Endosc.* 2023;37:S384.

Exclusion reason: Abstract only - insufficient data

Ikramuddin S, Korner J, Lee WJ, et al. Bariatric Surgery and Diabetes. *Diabetes Technol Ther.* 2015;17:S76-S79.

Exclusion reason: Abstract only - insufficient data

Ito CS, King SL, Nakagawa K. Ethnic disparities in the use of tissue plasminogen activator among young adult stroke patients in Hawaii. *Stroke.* 2016;47.

Exclusion reason: Abstract only - insufficient data

Margulies S, Sakai N, Geller E. Racial Disparities in National Practice Patterns for Stress Urinary Incontinence Surgery. *Int Urogynecol J.* 2022;33:S464.

Exclusion reason: Abstract only - insufficient data

Misra R, Lambert L, Vera D, Mangaraj A, Khanna SR, Sen CK. Wound healing by racial/ethnic categories. *Wound Repair Regen.* 2010;18(2):A46.

Exclusion reason: Abstract only - insufficient data

Nair BG, Espina C, Horibe M, et al. Evaluation of a continuous glucose monitor during and after surgery. *Diabetes.* 2017;66:A243.

Exclusion reason: Abstract only - insufficient data

Pandher M, Malhotra R, Alpert E, Casey S, Abedin Y. The effect of metabolic syndrome on postoperative morbidity and mortality of patients with endometrial cancer: An ACS-NSQIP study. *Gynecol Oncol.* 2022;166:S207-S208.

Exclusion reason: Abstract only - insufficient data

Park H, Jeong D, Nguyen P, et al. The increasing burden of hospital charges from viral hepatitis in the United States (US) between 2005 and 2013: Result of a population-based cohort of 85,926 HBV and 539,438 HCV patients. *Hepatol Int.* 2017;11(1):S129.

Exclusion reason: Abstract only - insufficient data

Wang KM, Isom RT. Euglycemic ketoacidosis in a post-operative patient taking empagliflozin. *Am J Kidney Dis* 2019;73(5):745.

Exclusion reason: Abstract only - insufficient data

O'Brien PE. Bariatric surgery: Mechanisms, indications and outcomes. *J Gastroenterol Hepatol.* 2010;25(8):1358-1365.

Exclusion reason: Non-US Study, study conducted in Australia

Luo M, Zhou E, Peng F. Type 2 diabetes mellitus increases postoperative recurrence risk in Chinese patients with chronic rhinosinusitis. *Acta Otolaryngol.* 2023;143(9):783-788.

Exclusion reason: Non-US Study, study conducted in China

Shih K-C, Janckila AJ, Lee W-J, et al. Effects of bariatric weight loss surgery on glucose metabolism, inflammatory cytokines, and serum tartrate-resistant acid phosphatase 5a in obese Chinese adults. *Clin Chim Acta.* 2016;453:197-202.

Exclusion reason: Non-US Study, study conducted in China

Su W, Tai S, Huang Y, Hu X, Zhou S, Fang Z. Clinical characteristics of severe aortic stenosis patients combined with diabetes mellitus after transcatheter aortic valve replacement and short-term outcome. Z*hong Nan Da Xue Xue Bao Yi Xue Ban.* 2022;47(3):309-318.

Exclusion reason: Non-US Study, study conducted in China

Tian L, Zhu J, Liu L, Liang Y, Li J, Yang Y. Hemoglobin A1c and short-term outcomes in patients with acute myocardial infarction undergoing primary angioplasty: an observational multicenter study. *Coron Artery Dis.* 2013;24(1):16-22.

Exclusion reason: Non-US Study, study conducted in China

Yi B, Jiang J, Zhu L, Li P, Im I, Zhu S. Comparison of the effects of Roux-en-Y gastrojejunostomy and LRYGB with small stomach pouch on type 2 diabetes mellitus in patients with BMI <35 kg/m^2^. *Surg Obes Relat Dis.* 2015;11(5):1061-1068.

Exclusion reason: Non-US Study, study conducted in China

Yu X, Huang Y-H, Feng Y-Z, Cheng Z-Y, Wang C-C, Cai X-R. Association of body composition with postoperative complications after laparoscopic sleeve gastrectomy and Roux-en-Y gastric bypass. *Eur J Radiol.* 2023;162.

Exclusion reason: Non-US Study, study conducted in China

Chan VWK, Chan PK, Woo YC, et al. Universal haemoglobin A1c screening reveals high prevalence of dysglycaemia in patients undergoing total knee arthroplasty. *Hong Kong Med J.* 2020;26(4):304-310.

Exclusion reason: Non-US Study, study conducted in Hong Kong

Bano T, Kuchay MS, Mishra SK, et al. Immediate postoperative complications following coronary artery bypass grafting in patients with type 2 diabetes: A prospective cohort study. *Diabetes Metab Syndr.* 2020;14(1):47-51.

Exclusion reason: Non-US Study, study conducted in India

Enas EA, Senthilkumar A, Vinod C, Puthumana N. Dyslipidaemia among Indo-Asians strategies for identification and management. *Br J Diabetes Vasc Dis.* 2005;5(2):81-90.

Exclusion reason: Non-US Study, study conducted in India

Kumar P, Renuka MK, Kalaiselvan MS, Arunkumar AS. Outcome of Noncardiac Surgical Patients Admitted to a Multidisciplinary Intensive Care Unit. *Indian J Crit Care Med.* 2017;21(1):17-22.

Exclusion reason: Non-US Study, study conducted in India

Shah SS, Todkar JS, Shah PS, Cummings DE. Diabetes remission and reduced cardiovascular risk after gastric bypass in Asian Indians with body mass index <35 kg/m^2^. *Surg Obes Relat Dis.* 2010;6(4):332-338.

Exclusion reason: Non-US Study, study conducted in India

Karim HMR, Sharma A, Khan TH, Abayadeera AU, Alam MR. A multinational online survey on the current perioperative practice for the management of patients with diabetes mellitus: a descriptive study. *Anaesth Pain Intensive Care.* 2022;26(3):382-392.

Exclusion reason: Non-US Study, study conducted in India, Pakistan, Bangaldesh, Nepal, Bhutan, and Sri Lanka

Ono T, Kobayashi J, Sasako Y, et al. The impact of diabetic retinopathy on long-term outcome following coronary artery bypass graft surgery. *J Am Coll Cardiol.* 2002;40(3):428-436.

Exclusion reason: Non-US Study, study conducted in Japan

Shiota M, Yokomizo A, Takeuchi A, et al. The feature of metabolic syndrome is a risk factor for biochemical recurrence after radical prostatectomy. *J Surg Oncol.* 2014;110(4):476-481.

Exclusion reason: Non-US Study, study conducted in Japan

Takahashi R, Kajita Y, Iwahori Y, Harada Y. The relationship between clinical outcomes of arthroscopic rotator cuff repair and hemoglobin A1c. *Asia Pac J Sports Med Arthrosc Rehabil Technol.* 2022;30:21-24.

Exclusion reason: Non-US Study, study conducted in Japan

Okabayashi T, Nishimori I, Maeda H, Yamashita K, Yatabe T, Hanazaki K. Effect of intensive insulin therapy using a closed-loop glycemic control system in hepatic resection patients: A prospective randomized clinical trial. *Diabetes Care.* 2009;32(8):1425-1427.

Exclusion reason: Non-US Study, study conducted in Japan

Goh GS, Yue WM, Guo CM, Tan SB, Chen JLT. Comparative Demographics and Outcomes of Minimally Invasive Transforaminal Lumbar Interbody Fusion in Chinese, Malays, and Indians. *Clin Spine Surg.* 2021;34(2):66-72.

Exclusion reason: Non-US Study, study conducted in Singapore

Hong P, Song YG, Paek S. Possible effects of agent orange and posttraumatic stress disorder on hyperglycemia in Korean veterans from the US-Vietnam war. *Medicine (Baltimore).* 2021;100(25):E26508.

Exclusion reason: Non-US Study, study conducted in South Korea

Tsai M-S, Wang Y-C, Wang H-H, Lee P-H, Jeng L-B, Kao C-H. Pre-existing diabetes and risks of morbidity and mortality after liver transplantation: A nationwide database study in an Asian population. *Eur J Intern Med.* 2015;26(6):433-438.

Exclusion reason: Non-US Study, study conducted in Taiwan

Kim KY, Anoushiravani AA, Chen KK, et al. Perioperative Orthopedic Surgical Home: Optimizing Total Joint Arthroplasty Candidates and Preventing Readmission. *J Arthroplasty.* 2019;34(7):S91-S96.

Exclusion reason: Not focusing on patients with either Type 2 diabetes mellitus or metabolic syndrome

Zhu D, Everly MJ. Deceased donor kidney transplantation in the United States from 1988 to 2011: an analysis of the OPTN/UNOS registry. *Clin Transpl.* 2012:1-12.

Exclusion reason: Not focusing on patients with either Type 2 diabetes mellitus or metabolic syndrome

Carter JS, Pugh JA, Monterrosa A. Non-insulin-dependent diabetes mellitus in minorities in the United States. *Ann Intern Med.* 1996;125(3):221-232.

Exclusion reason: Wrong outcomes

Greenfield DM, Salooja N, Peczynski C, et al. Metabolic syndrome and cardiovascular disease after haematopoietic cell transplantation (HCT) in adults: an EBMT cross-sectional non-interventional study. *Bone Marrow Transplant.* 2021;56(11):2820-2825.

Exclusion reason: Wrong outcomes

Gruenbaum SE, Guay CS, Gruenbaum BF, et al. Perioperative glycemia management in patients undergoing craniotomy for brain tumor resection: A global survey of neuroanesthesiologists' perceptions and practices. *World Neurosurg.* 2021;155:e548-e563.

Exclusion reason: Wrong outcomes

Hsu WC, Boyko EJ, Fujimoto WY, et al. Pathophysiologic differences among Asians, Native Hawaiians, and other Pacific Islanders and treatment implications. *Diabetes Care.* 2012;35(5):1189-1198.

Exclusion reason: Wrong outcomes

Ikramuddin S, Korner J, Lee WJ, et al. Roux-en-Y gastric bypass vs intensive medical management for the control of type 2 diabetes, hypertension, and hyperlipidemia: the Diabetes Surgery Study randomized clinical trial. *JAMA.* 2013;309(21):2240-2249.

Exclusion reason: Wrong outcomes

Ikramuddin S, Korner J, Lee WJ, et al. Lifestyle intervention and medical management with vs without roux-en-y gastric bypass and control of hemoglobin A1c, LDL cholesterol, and systolic blood pressure at 5 years in the Diabetes Surgery Study. *JAMA.* 2018;319(3):266-278.

Exclusion reason: Wrong outcomes

Joseph JJ, Ortiz R, Acharya T, Golden SH, Lopez L, Deedwania P. Cardiovascular impact of race and ethnicity in patients with diabetes and obesity JACC Focus Seminar 2/9. *J Am Coll Cardiol.* 2021;78(24):2471-2482.

Exclusion reason: Wrong outcomes

King GL, McNeely MJ, Thorpe LE, et al. Understanding and addressing unique needs of diabetes in Asian Americans, Native Hawaiians, and Pacific Islanders. *Diabetes Care.* 2012;35(5):1181-1188.

Exclusion reason: Wrong outcomes

Nasiri E, Mollaei A, Birami M, Lotfi M, Rafiei MH. The risk of surgery-related pressure ulcer in diabetics: A systematic review and meta-analysis. *Ann Med Surg.* 2021;65.

Exclusion reason: Wrong outcomes

Ritz E, Rychlik I, Locatelli F, Halimi S. End-stage renal failure in type 2 diabetes: A medical catastrophe of worldwide dimensions. *Am J Kidney Dis.* 1999;34(5):795-808.

Exclusion reason: Wrong outcomes

Xia JL, Patnaik JL, Lynch AM, Christopher KL. Comparison of cataract surgery outcomes in patients with type 1 vs type 2 diabetes mellitus and patients without diabetes mellitus. *J Cataract Refract Surg.* 2023;49(6):608-613.

Exclusion reason: Wrong outcomes

Azagury DE, Lautz DB. Obesity Overview: Epidemiology, Health and Financial Impact, and Guidelines for Qualification for Surgical Therapy. *Gastrointest Endosc Clin North Am.* 2011;21(2):189-201.

Exclusion reason: Wrong patient population

Grek S, Gravenstein N, Morey TE, Rice MJ. A cost-effective screening method for preoperative hyperglycemia. *Anesth Analg.* 2009;109(5):1622-1624.

Exclusion reason: Wrong patient population

Ikramuddin S, Korner J, Lee W-J, et al. Lifestyle Intervention and Medical Management With vs Without Roux-en-Y Gastric Bypass and Control of Hemoglobin A<sub>1c</sub>, LDL Cholesterol, and Systolic Blood Pressure at 5 Years in the Diabetes Surgery Study. *JAMA.* 2018;319(3):266-278.

Exclusion reason: Wrong patient population

Ledesma JA, Issa TZ, Lambrechts MJ, et al. Multilevel ossification of the posterior longitudinal ligament causing cervical myelopathy: An observational series of North American patients. *J Craniovertebr Junction Spine.* 2023;14(3):292-298.

Exclusion reason: Wrong patient population

Li C, Lin S, Liang H. Single-Anastomosis Duodenal Switch: Conceptual Difference between East and West. *Obes Surg.* 2021;31(7):3296-3302.

Exclusion reason: Wrong patient population

Liow MHL, Lee M, Goh GSH, et al. Poorer Fusion Outcomes in Diabetic Cervical Spondylotic Myelopathy Patients Undergoing Single-level Anterior Cervical Discectomy and Fusion Does Not Compromise Functional Outcomes and Quality of Life. *Spine.* 2018;43(7):477-483.

Exclusion reason: Wrong patient population

Mbata O, Abo El-Magd NF, El-Remessy AB. Obesity, metabolic syndrome and diabetic retinopathy: Beyond hyperglycemia. *World J Diabetes.* 2017;8(7):317-329.

Exclusion reason: Wrong patient population

Miki E, Lu M, Lee ET, Keen H, Bennett PH, Russell D. Risk factors, ethnic differences and mortality associated with lower-extremity gangrene and amputation in diabetes. The WHO multinational study of vascular disease in diabetes. *Diabetologia.* 2001;44(SUPPL. 2):S65-S71.

Exclusion reason: Wrong patient population

Miyatake K, Takeda Y, Fujii K, et al. Comparable clinical and structural outcomes after arthroscopic rotator cuff repair in diabetic and non-diabetic patients. *Knee Surg Sports Traumatol Arthroscopy.* 2018;26(12):3810-3817.

Exclusion reason: Wrong patient population

Moinuddin I, Yaqub MS, Taber T, Powelson J, Fridell J, Sharfuddin A. Isolated pancreas rejections do not have an adverse impact on kidney graft survival whereas kidney rejections are associated with adverse pancreas graft survival in simultaneous pancreas kidney transplantation. *J Nephrol.* 2018;31(2):307-315.

Exclusion reason: Wrong patient population

Morrish NJ, Wang SL, Stevens LK, Fuller JH, Keen H. Vascular disease in younger-onset diabetes: Comparison of European, Asian and American Indian cohorts of the WHO multinational study of vascular disease in diabetes. *Diabetologia.* 2001;44(SUPPL. 2):S78-S81.

Exclusion reason: Wrong patient population

Navaneethan SD, Schold JD, Srinivas TR. Metabolic Syndrome and Mild to Moderate Chronic Kidney Disease Among Minorities. *Semin Nephrol.* 2010;30(1):51-58.

Exclusion reason: Wrong patient population

Palepu S, Prasad GV. New-onset diabetes mellitus after kidney transplantation: Current status and future directions. *World J Diabetes.* 2015;6(3):445-455.

Exclusion reason: Wrong patient population

Schwartz SB, Rothrock M, Barron-Vaya Y, et al. Impact of Diabetes on Burn Injury: Preliminary Results From Prospective Study. *J Burn Care Res.* 2011;32(3):435-441.

Exclusion reason: Wrong patient population

Sermkasemsin V, Rungreungvanich M, Apinyachon W, Sangasilpa I, Srichot W, Pisitsak C. Incidence and risk factors of intraoperative hyperglycemia in non-diabetic patients: a prospective observational study. *BMC Anesthesiol.* 2022;22(1).

Exclusion reason: Wrong patient population

Takahashi R, Kajita Y, Harada Y, Iwahori Y, Deie M. Clinical results of arthroscopic rotator cuff repair in diabetic and non-diabetic patients. *J Ortop Sci.* 2021;26(2):213-218.

Exclusion reason: Wrong patient population

Wiznia DH, Jimenez R, Harrington M. Movement Is Life-Optimizing Patient Access to Total Joint Arthroplasty: Diabetes Mellitus Disparities. *J Am Acad Orthop Surg.* 2022;30(21):1017-1022.

Exclusion reason: Wrong patient population

Zhong Y, Cheng Y, Chen L, Yin Y. Benign breast disease with malignant imaging features: a case report. *J Med Case Rep.* 2023;17(1).

Exclusion reason: Wrong patient population

Phelan S, Kanaya AM, Subak LL, et al. Prevalence and risk factors for urinary incontinence in overweight and obese diabetic women: Action for Health in Diabetes (Look AHEAD) study. *Diabetes Care.* 2009;32(8):1391-1397.

Exclusion reason: Wrong setting

Huang Z-P, Guo Y, Liu C-Q, Qi L, Zou D-J, Zhou W-P. The effect of metabolic surgery on nonobese patients (BMI &lt; 30 kg/m<SUP>2</SUP>) with type 2 diabetes: a systematic review. *Surgery for Obesity and Related Diseases.* 2018;14(6):810-820.

Exclusion reason: Wrong study design

Kaur K, Joyner RW. Diabetes Intraoperative Management. *StatPearls Publishing.* 2022;01:01.

Exclusion reason: Wrong study design
